# Supplementary material for: Geometry and UV-Vis Spectra of Au3+ Complexes with Hydrazones Derived from Pyridoxal 5′-Phosphate: A DFT Study
Source: Int J Mol Sci. 2023 May 7;24(9):8412. doi: 10.3390/ijms24098412 (PMC10179053; doi:10.3390/ijms24098412)
Supplement: Supplementary file 1 [file ijms-24-08412-s001.zip › ijms-2384696-supplementary.pdf]

Supplementary Data for

Geometry and UV-Vis spectra of Au^{3+} complexes with hydrazones derived from pyridoxal 5′-phosphate: A DFT study

Oleg A. Pimenov[1], Konstantin V. Grazhdan[1], Maksim N. Zavalishin[1], and George A. Gamov[1,*]

Ivanovo 153000, Russia, Ivanovo, Sheremetevskii pr. 7, Ivanovo State University of Chemistry and Technology

* Correspondence: George A. Gamov, ggamov@isuct.ru; Tel.: +7(915)8218562

Table S1. Optimized geometry (*xyz*-coordinates) of different protonated species of complex formed by gold(III) and hydrazone derived from pyridoxal 5′-phosphate and 2-furoylhydrazide (**PLP-F2H**).

Deprotonated complex, $AuClL^{-}$
6 -1.755336000 -3.565942000 -0.123448000
6 -0.985221000 -2.359148000 -0.135928000
6 -1.661630000 -1.132413000 -0.334982000
6 -3.075878000 -1.169663000 -0.498426000
6 -3.705303000 -2.389679000 -0.462178000
7 -3.062383000 -3.557980000 -0.284180000
1 -4.779963000 -2.448445000 -0.581403000
6 -1.061227000 -4.879324000 0.077850000
1 -0.528943000 -4.900427000 1.030413000
1 -0.316176000 -5.053148000 -0.700550000
1 -1.794243000 -5.681420000 0.058338000
8 0.312632000 -2.543179000 0.042048000
6 -1.018124000 0.151355000 -0.387330000
1 -1.623258000 1.034753000 -0.540517000
6 -3.896263000 0.074796000 -0.706287000
1 -4.928291000 -0.202566000 -0.918602000
1 -3.528807000 0.644364000 -1.564965000
8 -3.841659000 0.880423000 0.477576000
15 -4.456150000 2.414480000 0.424145000
8 -5.727264000 2.410251000 -0.366502000
8 -4.366186000 2.925884000 1.827765000
8 -3.333986000 3.183688000 -0.510353000
7 0.248236000 0.370939000 -0.255778000
7 0.761956000 1.637597000 -0.321785000
6 2.056472000 1.644750000 -0.147713000
8 2.791872000 0.571253000 0.064797000
6 2.749847000 2.915616000 -0.186282000
6 2.318753000 4.198896000 -0.374600000
6 3.473211000 5.022654000 -0.304496000
1 1.301649000 4.505688000 -0.541252000
1 3.519096000 6.092526000 -0.407163000
8 4.103584000 2.901228000 -0.004192000
6 4.522657000 4.186822000 -0.079322000
79 1.605481000 -1.045894000 0.072154000
17 3.341682000 -2.554569000 0.457540000
1 -2.617286000 3.516017000 0.041808000
1 5.578515000 4.346159000 0.045341000
Monoprotonated complex, $AuClLH^{0}$
6 -1.667960000 -3.537187000 -0.124055000

6	-0.922173000	-2.328739000	-0.133948000
6	-1.607417000	-1.103411000	-0.346491000
6	-3.018560000	-1.126231000	-0.531182000
6	-3.676685000	-2.323351000	-0.505275000
7	-2.984346000	-3.467268000	-0.309895000
1	-4.741063000	-2.424182000	-0.633346000
6	-1.021218000	-4.858646000	0.084726000
1	-0.504592000	-4.873987000	1.044633000
1	-0.268603000	-5.031830000	-0.684843000
1	-1.750947000	-5.664085000	0.058407000
8	0.363835000	-2.514480000	0.059321000
6	-0.964622000	0.186729000	-0.393536000
1	-1.573141000	1.067292000	-0.538633000
6	-3.829448000	0.125260000	-0.757073000
1	-4.865814000	-0.142730000	-0.956973000
1	-3.455884000	0.665152000	-1.632700000
8	-3.745910000	0.941271000	0.407470000
15	-4.634633000	2.345870000	0.454133000
8	-5.980769000	2.082344000	-0.142884000
8	-4.434847000	2.881164000	1.835655000
8	-3.825489000	3.280803000	-0.630611000
7	0.301995000	0.401919000	-0.258481000
7	0.812866000	1.656783000	-0.322637000
6	2.113828000	1.663994000	-0.146980000
8	2.842908000	0.589007000	0.066644000
6	2.803250000	2.929589000	-0.186673000
6	2.365770000	4.213188000	-0.373880000
6	3.515307000	5.039270000	-0.306247000
1	1.346864000	4.515634000	-0.537556000
1	3.557784000	6.109186000	-0.408399000
8	4.157444000	2.919991000	-0.007646000
6	4.569169000	4.205625000	-0.083642000
79	1.661165000	-1.017904000	0.079698000
17	3.388409000	-2.519254000	0.471505000
1	5.624607000	4.370485000	0.038087000
1	-3.504855000	-4.337072000	-0.301108000
1	-3.066298000	3.702430000	-0.212243000
Bis-protonated complex, $AuClLH_2^+$			
6	-1.712243000	-3.527647000	-0.108713000
6	-0.962206000	-2.315899000	-0.114329000
6	-1.648942000	-1.093455000	-0.323877000
6	-3.059866000	-1.107110000	-0.509680000
6	-3.718051000	-2.303659000	-0.487820000
7	-3.024472000	-3.448293000	-0.295739000

1	-4.782144000	-2.405304000	-0.616762000
6	-1.068654000	-4.849591000	0.096620000
1	-0.554851000	-4.867889000	1.058063000
1	-0.314252000	-5.019504000	-0.672012000
1	-1.799972000	-5.652943000	0.065134000
8	0.321688000	-2.519825000	0.081603000
6	-1.003432000	0.199370000	-0.367095000
1	-1.627376000	1.075200000	-0.491922000
6	-3.864039000	0.148975000	-0.739823000
1	-4.914751000	-0.108771000	-0.860739000
1	-3.537484000	0.638333000	-1.663572000
8	-3.689737000	1.014493000	0.376036000
15	-4.594045000	2.414913000	0.444479000
8	-5.978632000	2.105218000	-0.026808000
8	-4.282800000	3.002734000	1.782344000
8	-3.887832000	3.310038000	-0.738914000
7	0.263714000	0.370539000	-0.241913000
7	0.860911000	1.600424000	-0.287939000
6	2.199324000	1.645948000	-0.134174000
8	2.851133000	0.556783000	0.049855000
6	2.883063000	2.892500000	-0.173301000
6	2.476460000	4.199224000	-0.325872000
6	3.644663000	4.983401000	-0.268179000
1	1.470322000	4.558818000	-0.458073000
1	3.715444000	6.053113000	-0.349200000
8	4.241112000	2.842462000	-0.025131000
6	4.680733000	4.110349000	-0.085815000
79	1.634248000	-1.051041000	0.078564000
17	3.318462000	-2.567080000	0.427629000
1	5.742797000	4.246971000	0.014300000
1	-3.548277000	-4.317396000	-0.290581000
1	-3.123010000	3.783261000	-0.392054000
1	0.283406000	2.416647000	-0.446405000

Table S2. Calculated IR spectra of different protonated species of complex formed by gold(III) and hydrazone derived from pyridoxal 5′-phosphate and 2-furoylhydrazide (**PLP-F2H**).

Deprotonated complex, $AuClL^-$		Monoprotonated complex, $AuClLH^0$		*Bis*-protonated complex, $AuClLH_2^+$	
Frequency, cm^{-1}	Relative intensity	Frequency, cm^{-1}	Relative intensity	Frequency, cm^{-1}	Relative intensity
17.6909	11.0545	17.8764	15.7848	19.309	0.4724

22.6886	6.8672	21.2555	18.4836	21.7872	23.3133
32.7716	5.8183	33.5437	2.9842	24.941	27.7322
41.1999	1.145	38.323	2.3226	36.5281	5.5634
46.4176	0.4316	51.2607	0.6803	55.7036	1.4575
60.0311	1.1877	61.5951	3.268	60.2754	5.1148
66.3806	0.7337	68.1117	1.2275	68.5156	0.7875
84.8173	0.4881	79.295	1.7555	79.5466	0.3489
98.7937	5.7411	99.5188	6.5661	97.969	10.5416
110.618	3.2276	110.7968	3.9888	110.1021	3.1759
115.055	2.9341	115.4898	1.2144	118.6565	4.2542
137.744	7.3175	135.4489	2.8848	137.4516	2.7545
155.008	1.4463	146.6584	14.697	146.0282	20.6633
160.088	11.4922	160.8298	4.6755	158.6945	1.8928
185.48	2.9921	183.6845	7.4802	174.1607	12.3223
188.034	3.2809	188.1552	3.9888	182.7982	4.2712
205.39	0.588	206.8566	1.5191	189.6276	5.3221
224.378	12.5999	223.4913	13.1813	216.4898	15.1568
230.951	2.6194	228.7327	6.1176	221.5302	1.6159
244.303	6.6572	246.1331	6.921	240.6859	8.0767
261.844	5.6616	258.3886	4.1059	249.1301	2.3458
290.306	65.1325	292.1205	56.2142	295.2806	38.0929
326.464	72.8411	325.6133	21.8915	322.8712	20.9982
328.072	14.7081	334.3338	58.9992	329.9869	1.8576
340.655	3.2564	336.6326	3.8579	338.4246	2.0728
348.724	4.9706	346.1875	5.3108	355.219	41.2874
373.662	40.2934	370.2253	19.2567	360.8923	19.058
397.367	49.1008	403.9631	79.581	402.4868	29.1045
418.801	22.7313	420.1515	17.0656	405.1796	81.5411
435.772	0.7551	439.6133	4.7304	419.4093	8.9909
441.818	2.7304	457.5576	3.6902	435.7666	11.892
466.15	110.972	466.9646	95.2873	445.2133	98.4745
477.716	55.5234	478.6086	82.1814	466.1097	112.1095
490.285	60.4583	486.5737	44.5857	477.7534	73.6825
503.209	38.8364	501.5035	16.1955	485.2528	30.5422
541.313	85.3339	538.7234	74.6098	496.5519	34.098
578.376	21.3066	565.5252	0.9493	534.7723	76.9673
581.447	10.0452	580.7774	1.2696	564.2412	7.3233
608.527	12.7688	605.6699	13.2376	572.8338	10.0887
616.444	42.5908	617.1217	17.401	594.5861	24.0902
631.478	9.3406	630.6131	12.053	613.8171	27.5643

637.602	83.9238	637.4639	72.4451	619.5573	5.7615
653.41	18.1453	659.6379	22.8973	632.9964	86.7764
684.538	29.023	686.5032	25.5702	649.7541	43.553
743.822	16.9044	746.2153	20.1905	676.965	11.0971
757.389	8.7215	756.9044	148.3992	739.1897	19.4809
762.126	115.209	763.1575	86.4467	749.696	179.8744
773.21	311.249	773.9151	27.0225	759.6703	128.098
776.064	86.4201	782.7502	290.2559	778.7463	16.9936
787.668	40.3456	783.0056	82.9256	785.1473	276.019
826.633	124.157	793.6823	94.7163	799.153	109.1222
864.888	5.5602	842.6096	135.5156	800.2444	67.9824
896.698	9.0471	872.6317	3.7383	845.583	141.6761
903.693	83.0406	896.4333	10.0961	888.1839	5.5983
921.764	1.2664	903.4978	127.3325	895.5776	0.328
922.982	39.7234	914.2202	14.8301	903.0274	94.9219
953.776	7.8024	928.6613	1.016	923.2609	7.6296
958.807	59.81	936.9425	12.509	946.5588	0.9628
976.403	37.0491	961.0614	40.5617	951.043	10.6022
1007.16	61.1961	987.8222	55.9198	960.4256	164.5524
1020	188.21	1019.104	38.0874	989.9023	7.553
1026.41	165.019	1024.576	140.5597	1020.828	68.7997
1032.18	333.512	1033.586	275.3831	1024.167	122.5064
1041.38	19.7546	1043.074	330.666	1047.041	373.1595
1054	1.6394	1053.227	138.1687	1057.9	10.3966
1071.25	8.4987	1056.825	6.9523	1061.721	122.8383
1090.36	244.057	1082.805	16.8725	1068.873	68.4143
1095.89	21.9683	1095.265	256.6254	1076.772	42.0244
1108.5	46.2087	1099.525	16.5304	1097.771	194.3932
1156.22	230	1113.828	34.422	1118.444	126.8944
1200.92	15.1663	1158.018	498.0712	1123.928	247.0955
1217.66	381.568	1209.169	156.2511	1175.6	257.3497
1253.96	14.5013	1227.149	213.7499	1218.575	115.9398
1258.46	28.9546	1251.452	8.2326	1220.559	240.4853
1279.56	456.794	1259.049	28.9955	1250.492	6.3437
1290.12	195.809	1270.78	292.4325	1264.388	175.9775
1315.43	35.0572	1285.013	422.4333	1281.218	22.9897
1331.71	108.066	1329.526	29.5211	1289.413	410.9263
1349.54	268.229	1332.106	37.1153	1324.534	120.1843
1391.12	153.743	1363.105	393.2826	1334.867	123.1466
1399.71	42.7286	1388.068	173.9289	1365.25	96.0322

1407.35	19.9284	1400.862	98.9496	1388.355	122.8897
1413.17	13.279	1409.073	18.1833	1395.705	19.9858
1425.2	50.1983	1422.254	3.4312	1407.524	197.787
1464.99	23.5233	1429.775	20.2015	1420.281	21.143
1466.27	13.3927	1437.694	98.6115	1435.322	128.8015
1485.15	13.7675	1452.949	16.3125	1436.569	106.6888
1500.52	383.991	1481.617	666.9948	1450.641	384.9746
1519.26	11.7844	1489.993	1187.803	1452.277	17.3447
1542.8	717.978	1507.42	189.2682	1483.869	22.6041
1555.33	209.333	1525.328	77.7461	1501.672	271.6999
1598.89	9.6387	1533.209	615.0443	1520.257	413.1629
1611.35	152.362	1595.704	228.1379	1525.72	65.7742
1652.75	215.107	1622.304	57.3594	1561.818	566.1735
3014.65	44.2193	1649.5	58.0284	1616.595	1019.043
3034.76	23.2788	1669.954	74.8969	1621.781	412.9726
3076.33	46.2641	3012.919	39.9944	1661.641	51.7513
3080.4	17.5987	3048.563	2.6232	1684.016	20.8486
3133.27	23.5616	3086.521	30.5637	3007.349	35.8629
3163.98	48.0733	3102.815	0.8995	3049.514	1.6459
3189.96	42.8736	3137.492	16.883	3092.428	23.4647
3253.87	4.1399	3212.032	45.4361	3104.085	0.1656
3267.81	1.5704	3253.752	3.095	3141.902	13.3911
3280.9	0.2781	3256.154	3.3523	3187.526	102.0188
3811.47	86.3335	3269.043	3.1608	3252.464	1.2169
		3281.711	0.5228	3257.642	7.9574
		3547.301	399.6079	3267.677	6.0399
		3810.588	88.1091	3284.424	4.2584
				3535.975	352.4969
				3562.918	161.622
				3810.186	94.4044

Table S3. Calculated TD-DFT spectra of different protonated species of complex formed by gold(III) and hydrazone derived from pyridoxal 5′-phosphate and 2-furoylhydrazide (**PLP-F2H**)

Deprotonated complex, $AuClL^-$		Monoprotonated complex, $AuClLH^0$		*Bis*-protonated complex, $AuClLH_2^+$	
λ, nm	Relative intensity	λ, nm	Relative intensity	λ, nm	Relative intensity
482.95	0	450.81	0	457.18	0.0001
373.72	0.0001	390.63	0.5444	376.13	0.0907
371.03	0.3073	349.66	0	367.36	0
317.39	0.0271	328.31	0.0723	362.11	0.2398

312.89	0.0001	328.19	0.0108	322.43	0.0007
308.57	0.5777	316.81	0.3263	305.77	0.7109
292.95	0.0002	295.46	0	300.09	0.0003
289.45	0.0026	283.97	0.0381	297.1	0.0017
279.51	0.0384	273.61	0.0012	285.92	0.0485
265.07	0.3953	268.15	0.134	283.73	0.0054
253.16	0.2016	266.52	0.0035	282.93	0.0271
250.79	0.0012	260.14	0.0466	272.92	0.0343
242.45	0.0016	258.89	0.1376	266.12	0.1248
240.01	0.0285	251.58	0.0005	262.97	0.007
238.88	0.0002	247.36	0.0006	260.09	0.0378
236.24	0.0137	240.82	0.3722	252.59	0.1219
230.52	0.0352	237.86	0.0007	245.03	0.1326
228.74	0.0659	232.45	0.0156	243.31	0.0362
224.41	0.0112	232.12	0.0481	241.7	0.0001
223.83	0.0673	230.8	0.0019	236.44	0.0008
220.91	0.0057	229.77	0.0334	230.12	0
219.78	0.0049	225.73	0.0001	228	0.0249
216.86	0.0714	225.24	0.0066	224.23	0.0866
214.44	0.0058	220.99	0.052	222.93	0.0011
212	0.0003	219.16	0.002	222.61	0.0069
211.06	0.1364	217.47	0.0042	221.9	0.001
206.48	0.0069	216.23	0	219.38	0.0703
206.44	0.0173	214.85	0.1168	218.02	0.0004
205.74	0.0741	208.47	0.0952	212.11	0.0258
204.49	0.0022	206.99	0.0001	209.91	0.1097

Table S4. Optimized geometry (*xyz*-coordinates) of different protonated species of complex formed by gold(III) and hydrazone derived from pyridoxal 5′-phosphate and 2-methyl-3-furoylhydrazide (**PLP-F3H**)

Deprotonated complex, $AuClL^{-}$
6 -2.223033000 -3.443392000 -0.091260000
6 -1.335102000 -2.320517000 -0.113116000
6 -1.886843000 -1.031458000 -0.304852000
6 -3.297479000 -0.927777000 -0.471090000
6 -4.045578000 -2.078965000 -0.431959000
7 -3.523257000 -3.304936000 -0.247528000
1 -5.120312000 -2.030629000 -0.554707000
6 -1.664179000 -4.818940000 0.115970000

1	-1.128844000	-4.886584000	1.064676000
1	-0.946952000	-5.073679000	-0.666289000
1	-2.475017000	-5.542534000	0.108836000
8	-0.060929000	-2.634088000	0.052276000
6	-1.120131000	0.183822000	-0.345326000
1	-1.639474000	1.123340000	-0.475708000
6	-3.990724000	0.388522000	-0.699771000
1	-5.044078000	0.210270000	-0.913088000
1	-3.565126000	0.905663000	-1.565166000
8	-3.862563000	1.207020000	0.469288000
15	-4.436297000	2.757635000	0.418149000
8	-5.745044000	2.776301000	-0.308653000
8	-4.263318000	3.286917000	1.807275000
8	-3.344705000	3.481182000	-0.584379000
7	0.162053000	0.278128000	-0.224649000
7	0.795768000	1.491433000	-0.276200000
1	-2.579983000	3.781306000	-0.080421000
6	2.090595000	1.377030000	-0.137698000
8	2.711657000	0.222052000	0.033778000
6	2.899541000	2.582931000	-0.175389000
6	2.397683000	3.914848000	-0.403358000
6	3.466186000	4.731359000	-0.359757000
1	1.373865000	4.193344000	-0.574640000
1	3.598326000	5.791835000	-0.470713000
6	4.258182000	2.699068000	-0.006899000
6	5.369967000	1.755741000	0.257132000
1	4.988837000	0.753507000	0.415640000
1	6.065577000	1.738666000	-0.584851000
1	5.930932000	2.069901000	1.139067000
8	4.604831000	4.005815000	-0.118549000
79	1.376266000	-1.270842000	0.051395000
17	2.969753000	-2.946469000	0.368208000
Monoprotonated complex, $AuClLH^0$			
6	-2.308614000	-3.370286000	-0.078936000
6	-1.411327000	-2.269499000	-0.106597000
6	-1.936833000	-0.963514000	-0.292737000
6	-3.342942000	-0.802872000	-0.446813000
6	-4.151489000	-1.903738000	-0.403869000
7	-3.608830000	-3.128640000	-0.225668000
1	-5.222621000	-1.864614000	-0.506612000
6	-1.835508000	-4.765830000	0.110158000
1	-1.316938000	-4.858446000	1.064873000
1	-1.118109000	-5.025649000	-0.668319000
1	-2.663311000	-5.470159000	0.083097000
8	-0.156682000	-2.624757000	0.047683000

6	-1.133489000	0.234723000	-0.342160000
1	-1.624532000	1.188574000	-0.474557000
6	-3.987613000	0.543353000	-0.665518000
1	-5.050142000	0.409789000	-0.864435000
1	-3.551595000	1.027862000	-1.543920000
8	-3.798248000	1.341783000	0.499338000
15	-4.190993000	2.954387000	0.412861000
8	-5.441223000	3.099688000	-0.394922000
8	-4.040162000	3.469249000	1.807892000
8	-2.961263000	3.527844000	-0.523079000
7	0.151366000	0.284108000	-0.225209000
7	0.817729000	1.466742000	-0.278134000
6	2.115253000	1.312131000	-0.143069000
8	2.695796000	0.138459000	0.025699000
6	2.959431000	2.485590000	-0.184627000
6	2.512260000	3.830331000	-0.448119000
6	3.610124000	4.605364000	-0.397615000
1	1.503570000	4.143683000	-0.646574000
1	3.786828000	5.657354000	-0.524644000
6	4.320265000	2.553473000	0.011549000
6	5.386672000	1.569770000	0.310387000
1	4.961259000	0.641309000	0.675075000
1	5.970394000	1.350534000	-0.587158000
1	6.069357000	1.975520000	1.057101000
8	4.715679000	3.840405000	-0.116799000
79	1.323297000	-1.304898000	0.045699000
17	2.860405000	-3.019303000	0.359030000
1	-4.236973000	-3.923524000	-0.197680000
1	-2.219828000	3.796228000	0.031488000
Bis-protonated complex, $AuClLH_2^+$			
6	-2.321531000	-3.364784000	-0.076100000
6	-1.430217000	-2.252828000	-0.103627000
6	-1.970517000	-0.953504000	-0.276776000
6	-3.378036000	-0.797819000	-0.421717000
6	-4.176419000	-1.905212000	-0.377724000
7	-3.620179000	-3.126455000	-0.210641000
1	-5.248648000	-1.875866000	-0.470691000
6	-1.836469000	-4.756387000	0.100341000
1	-1.309890000	-4.849359000	1.050704000
1	-1.122921000	-5.004192000	-0.685603000
1	-2.659162000	-5.466221000	0.074821000
8	-0.175399000	-2.616692000	0.039402000
6	-1.175995000	0.254866000	-0.320082000
1	-1.687599000	1.202208000	-0.442187000

6	-4.029667000	0.545846000	-0.636912000
1	-5.101050000	0.412264000	-0.776865000
1	-3.638877000	1.003311000	-1.551117000
8	-3.772450000	1.369903000	0.495104000
15	-4.180694000	2.982031000	0.390040000
8	-2.972924000	3.531877000	-0.589274000
8	-5.455048000	3.092304000	-0.383403000
8	-3.989387000	3.529192000	1.766663000
7	0.103484000	0.271855000	-0.201762000
7	0.845882000	1.421584000	-0.236256000
1	0.366458000	2.306950000	-0.334159000
6	2.184102000	1.311515000	-0.116021000
8	2.696874000	0.142838000	0.039521000
6	3.005486000	2.472197000	-0.165919000
6	2.616182000	3.844384000	-0.388721000
6	3.753655000	4.556515000	-0.349232000
1	1.631791000	4.242559000	-0.562295000
1	3.983430000	5.599509000	-0.459981000
6	4.382877000	2.468431000	-0.006657000
6	5.383011000	1.408639000	0.243669000
1	5.134404000	0.848489000	1.145009000
1	5.404337000	0.697152000	-0.582701000
1	6.367976000	1.853915000	0.356445000
8	4.827275000	3.725000000	-0.116166000
1	-2.277885000	3.932991000	-0.055345000
79	1.301185000	-1.310676000	0.049047000
17	2.806100000	-3.018830000	0.332262000
1	-4.243897000	-3.926043000	-0.181074000

Table S5. Calculated IR spectra of different protonated species of complex formed by gold(III) and hydrazone derived from pyridoxal 5′-phosphate and 2-methyl-3-furoylhydrazide (**PLP-F3H**).

Deprotonated complex, $AuClL^-$		Monoprotonated complex, $AuClLH^0$		*Bis*-protonated complex, $AuClLH_2^+$	
Frequency, cm^{-1}	Relative intensity	Frequency, cm^{-1}	Relative intensity	Frequency, cm^{-1}	Relative intensity
16.0312	8.9511	17.1566	6.3614	22.0751	6.462
17.8459	0.7559	22.6364	13.7083	28.7401	24.0671
25.0209	0.9898	25.7579	1.5337	40.2395	5.6366
33.9919	8.7845	31.0152	14.7946	44.0126	13.6275
42.9143	2.4071	40.8162	0.4603	49.3915	2.0872
55.6114	2.0418	46.4509	2.0305	60.9209	0.6931
66.0224	0.6836	64.0758	1.7271	65.5532	6.3568
67.3778	0.9909	71.4184	0.4093	78.8812	0.4205

94.2547	1.4068	95.5722	3.0097	93.9794	6.5635
99.7786	4.4729	100.0147	4.2274	96.6932	3.0534
111.8072	4.1389	110.2962	2.5627	109.2574	2.0544
116.3439	1.6991	117.4906	1.907	122.139	4.2226
142.8648	8.578	138.0872	3.0736	138.2962	3.0557
157.1403	4.1594	144.8027	3.3722	150.8365	5.1737
160.3637	5.5753	159.5208	17.607	156.4673	11.2981
175.6503	0.8224	176.0871	2.3452	168.3541	9.1726
188.6722	0.6149	185.9481	2.9319	182.252	4.1804
204.4101	1.9966	205.6836	5.7762	202.4022	6.8701
218.6113	2.3194	219.0531	4.5237	215.0035	6.7971
223.9216	5.431	227.2487	6.6687	220.7094	8.4155
235.3351	13.4218	236.0555	16.5414	238.069	9.0879
240.6256	14.427	241.3168	10.9347	245.3412	10.6012
284.0522	68.2088	293.2396	41.034	291.5557	0.1062
295.4671	1.5463	294.7699	0.3599	299.47	4.705
314.0681	8.4174	316.1039	21.2592	327.9072	1.7568
325.8393	77.7545	331.5628	55.9305	336.1479	12.6901
336.6753	9.2404	333.4745	21.1488	342.0473	13.4399
346.7685	5.5979	345.9219	7.2122	346.0153	28.0206
356.1987	1.2362	356.1621	5.9592	358.4859	17.8691
372.5896	35.9299	371.5093	33.0878	374.9796	3.7184
397.9047	47.8779	396.8241	73.4635	417.7715	21.7846
437.7503	32.8232	439.5706	36.091	438.7944	12.4005
443.1852	2.4409	439.9865	1.3515	443.1096	18.0119
443.7134	1.0131	464.496	30.0914	461.6319	168.5877
469.1266	94.013	469.4618	44.852	470.996	61.6008
482.3627	57.629	480.2516	61.8418	483.575	30.0883
490.543	58.6326	488.9648	65.2367	491.0846	79.2823
511.4101	43.4611	510.9932	25.4678	506.2753	31.5753
541.8906	97.4846	539.3482	84.6796	529.2574	98.0994
579.0061	13.5047	566.8423	0.4688	540.1303	85.9127
580.498	9.244	578.6695	0.1162	566.92	21.2187
607.2103	46.0977	608.6458	15.6745	572.1386	3.6606
617.1604	11.2852	616.2267	12.6425	602.5627	33.5114
636.1113	76.1684	637.333	61.0502	611.5252	2.666
647.8304	4.0182	647.5087	6.9978	634.3624	77.6083
650.6545	2.4487	654.1899	2.7395	642.3853	10.339
683.0607	50.16	685.4793	34.8405	656.9625	4.2336
700.3468	19.5046	703.4396	15.1905	686.4173	16.1264

746.7935	81.0503	749.2983	81.0229	702.1122	14.8989
758.2955	3.99	757.5211	139.8744	746.68	78.7462
762.7618	125.1803	763.6255	98.658	753.8677	227.091
775.3353	300.2802	774.0812	17.9732	764.5136	129.3234
775.8379	14.6961	775.5198	304.9272	772.2303	222.8103
787.8512	49.885	779.6871	1.2776	772.4144	34.2821
827.9487	130.775	793.7248	84.9749	779.7326	0.8573
888.5376	2.1211	841.7204	119.8066	799.9388	88.2176
904.7793	25.3767	891.1687	1.952	844.9914	121.4962
922.2061	60.5014	904.0873	47.3779	887.9916	0.1027
923.3588	43.0042	914.6803	11.3742	900.6633	25.0063
955.6526	7.5913	922.9224	78.555	920.6145	1.9265
964.8003	81.967	945.2079	9.6052	923.9012	6.5694
974.3587	45.9352	968.1004	84.4382	958.2431	9.5349
1005.061	57.3472	987.6164	59.0826	968.8764	274.7712
1019.333	154.3267	1013.612	38.7452	990.4843	30.3888
1025.646	108.5224	1019.12	142.6891	1018.7788	70.0912
1031.444	264.3784	1032.196	63.3035	1032.5319	84.4654
1054.086	3.6621	1044.219	297.5536	1047.8965	352.5151
1054.56	83.7815	1052.471	217.0672	1051.2491	52.1752
1063.421	7.3799	1056.954	8.1769	1054.6363	1.2121
1071.692	15.0576	1063.959	41.1353	1058.0315	8.6669
1091.349	196.8697	1081.877	31.2079	1061.7941	149.9798
1097.085	58.5088	1093.802	170.7419	1078.5245	46.0054
1143.168	115.1785	1099.21	91.4375	1096.7007	243.242
1148.034	249.0587	1144.21	21.8785	1108.9597	64.8408
1189.63	108.1383	1149.755	580.0483	1152.9397	60.0163
1216.399	365.9352	1196.269	245.8426	1181.0294	178.3118
1243.547	94.1493	1226.043	195.4323	1217.976	231.2445
1255.151	22.6882	1249.931	184.6328	1219.051	248.225
1279.273	447.0801	1257.221	9.2978	1256.7065	2.8801
1290.678	165.9575	1267.661	139.7586	1265.7651	243.7223
1316.812	44.391	1287.463	443.4717	1277.2952	169.5786
1331.587	107.8083	1330.315	56.1714	1291.8891	429.9208
1351.978	95.7495	1332.439	15.9497	1327.0689	85.9711
1390.766	128.3461	1360.713	142.6658	1336.9602	96.903
1398.115	9.9711	1389.102	189.9178	1363.0456	28.1908
1406.438	13.3977	1401.862	17.4709	1388.9963	105.6064
1409.184	6.5523	1407.82	83.5466	1400.5123	58.0083
1413.95	0.6844	1413.027	7.0272	1406.3688	79.2404

1457.377	202.4694	1425.328	12.914	1415.9753	32.084
1465.408	11.1777	1436.368	53.4933	1420.0635	240.7762
1465.946	25.6206	1452.726	16.6958	1425.8929	83.2705
1466.653	10.6086	1458.574	73.0581	1436.1108	107.976
1473.262	11.0602	1463.452	194.2151	1452.3789	17.6367
1485.914	3.829	1471.878	295.2273	1458.9255	17.2253
1522.75	27.3942	1479.974	539.2851	1476.1106	5.307
1527.591	781.8416	1499.643	509.304	1482.0202	25.4757
1554.093	63.5748	1523.02	91.884	1506.7213	56.4991
1563.016	154.584	1531.195	485.312	1523.3768	19.6017
1601.018	7.7751	1560.833	280.5323	1538.3569	426.8113
1621.615	419.4021	1599.341	326.4638	1545.5893	372.7101
1653.03	269.9376	1622.685	225.2205	1597.4215	568.4706
3011.437	45.0077	1650.739	92.8199	1599.8371	1215.9172
3033.11	31.3851	1669.573	80.6676	1619.88	0.615
3034.603	23.6433	3020.222	34.3505	1663.1414	39.5567
3077.929	13.8406	3032.969	17.9644	1684.2528	23.0057
3079.005	41.5036	3048.049	2.4866	3016.6171	30.0979
3080.139	17.768	3082.435	35.9391	3047.9624	1.8503
3132.883	23.9289	3087.916	13.5955	3049.2466	1.5593
3164.512	48.8224	3102.318	0.9572	3088.9257	29.5357
3167.56	0.8242	3137.105	16.3667	3100.1187	1.3326
3194.136	34.4074	3162.313	2.011	3103.7277	0.2054
3265.012	3.3621	3198.3	70.6782	3142.0505	13.3652
3286.871	0.5203	3249.374	2.5433	3142.9519	14.4906
3812.251	87.0589	3266.719	5.1371	3176.5726	130.2005
		3289.124	0.3932	3253.5366	7.0736
		3546.913	366.4172	3256.805	4.8371
		3810.009	92.9271	3295.9407	5.3802
				3537.6706	331.2896
				3593.1031	177.0285
				3813.4785	104.3756

Table S6. Calculated TD-DFT spectra of different protonated species of complex formed by gold(III) and hydrazone derived from pyridoxal 5′-phosphate and 2-methyl-3-furoylhydrazide (**PLP-F3H**)

Deprotonated complex, $AuClL^-$		Monoprotonated complex, $AuClLH^0$		*Bis*-protonated complex, $AuClLH_2^+$	
λ, nm	Oscillator strength	λ, nm	Oscillator strength	λ, nm	Oscillator strength
475.5	0	441.99	0.0002	452.87	0.0001
371.27	0.0004	382.48	0.4084	373.63	0.0671
365.64	0.2094	349.97	0	367.31	0.0003
316.08	0.028	329.93	0.0071	359.36	0.178
315.21	0.0022	325.85	0.0629	337.31	0.0004
301.07	0.538	315.34	0.2776	310.78	0.3531
299.93	0.0048	305.89	0.0001	302.19	0.0001
288.5	0.0017	290.05	0.0352	293.4	0.0007
280.35	0.0376	284.01	0.0919	285.78	0.0034
265.91	0.1942	271.53	0.0044	283.82	0.0224
258.4	0.1568	268.8	0.0005	279.81	0.0004
255.42	0.0051	259.33	0.1402	272.37	0.0682
243.48	0.0007	259.24	0.0266	265.37	0.0112
238.68	0.1755	248.59	0.0021	261.84	0.0429
237.35	0.0029	244.66	0.0001	258.97	0.0185
236.45	0.0207	243.84	0.0001	254.81	0.3824
229.39	0.121	237.52	0.0003	250.04	0.0076
227.96	0.005	234.29	0.4562	244.27	0.1631
226.41	0.078	231.07	0.0289	241.38	0.0007
221.53	0.0006	229.53	0.0101	236.41	0.001
219.37	0.0154	227.82	0.0746	230.58	0.1238
217.31	0.0007	227.21	0.007	229.2	0.0542
217.06	0.0068	223.55	0.0018	226.48	0.0026
214.19	0.0078	223.19	0.1204	223.25	0.1696
213.14	0.2779	218.68	0.0484	220.95	0.025
210.3	0.1835	217.26	0.038	220.14	0.0012
207.27	0.1184	216.87	0.0009	217.69	0.0549
206.1	0.0019	213.84	0.1109	217.31	0.0142
205.9	0.0002	207.8	0.0203	210.13	0.0648
203.97	0.0569	206.78	0.2248	208.71	0.0403

Table S7. Optimized geometry (*xyz*-coordinates) of different protonated species of complex formed by gold(III) and hydrazone derived from pyridoxal 5′-phosphate and thiophene-2-carbohydrazide (**PLP-T2H**)

Deprotonated complex, $AuClL^{-}$
6 -2.344734000 -3.370795000 -0.092215000
6 -1.414699000 -2.282538000 -0.114223000
6 -1.916899000 -0.973304000 -0.305039000
6 -3.322247000 -0.815944000 -0.474710000
6 -4.113478000 -1.937877000 -0.436407000
7 -3.638439000 -3.182603000 -0.249979000
1 -5.185312000 -1.848942000 -0.561567000
6 -1.839261000 -4.766583000 0.116406000
1 -1.304605000 -4.853129000 1.063936000
1 -1.134548000 -5.050509000 -0.667190000
1 -2.677682000 -5.458076000 0.112575000
8 -0.153346000 -2.645644000 0.049900000
6 -1.105027000 0.211789000 -0.340251000
1 -1.588197000 1.170567000 -0.468871000
6 -3.964635000 0.524736000 -0.710416000
1 -5.023897000 0.385220000 -0.923084000
1 -3.519298000 1.020024000 -1.578784000
8 -3.805972000 1.346106000 0.452773000
15 -4.356230000 2.905645000 0.401231000
8 -5.677221000 2.940293000 -0.302436000
8 -4.151006000 3.440153000 1.783883000
8 -3.272627000 3.605789000 -0.625520000
7 0.179666000 0.256251000 -0.214546000
7 0.859139000 1.443884000 -0.263371000
6 2.146168000 1.275829000 -0.120884000
8 2.727724000 0.100898000 0.051352000
6 3.025822000 2.429406000 -0.149081000
6 2.690183000 3.752074000 -0.317619000
6 3.811933000 4.609677000 -0.291932000
1 1.672415000 4.082686000 -0.454097000
1 3.754232000 5.681270000 -0.406898000
16 4.743658000 2.239526000 0.043125000
6 4.986992000 3.933572000 -0.104721000
79 1.334424000 -1.340468000 0.059545000
17 2.856103000 -3.078671000 0.383490000
1 -2.488936000 3.887287000 -0.140319000
1 5.983809000 4.337150000 -0.045336000
Monoprotonated complex, $AuClLH^{0}$
6 -2.326868000 -3.343678000 -0.078340000

6	-1.410637000	-2.258980000	-0.108705000
6	-1.912638000	-0.944729000	-0.301065000
6	-3.314366000	-0.760277000	-0.468688000
6	-4.141463000	-1.847329000	-0.426629000
7	-3.621653000	-3.080112000	-0.235899000
1	-5.210772000	-1.790981000	-0.539727000
6	-1.880152000	-4.745926000	0.124871000
1	-1.361222000	-4.837896000	1.079393000
1	-1.169859000	-5.028352000	-0.652307000
1	-2.721545000	-5.434320000	0.107442000
8	-0.162439000	-2.635904000	0.050079000
6	-1.089368000	0.239340000	-0.341504000
1	-1.566471000	1.200376000	-0.467532000
6	-3.935570000	0.593487000	-0.708924000
1	-5.002281000	0.475497000	-0.894606000
1	-3.497008000	1.052727000	-1.600288000
8	-3.720280000	1.415338000	0.434356000
15	-4.309522000	2.970333000	0.407403000
8	-5.672907000	2.957077000	-0.207736000
8	-4.023825000	3.513814000	1.770301000
8	-3.312785000	3.675193000	-0.695870000
7	0.195633000	0.267165000	-0.216332000
7	0.881554000	1.437571000	-0.265080000
6	2.173565000	1.257544000	-0.120751000
8	2.738008000	0.076710000	0.050825000
6	3.058869000	2.399020000	-0.147094000
6	2.726083000	3.725040000	-0.313721000
6	3.849346000	4.576612000	-0.287107000
1	1.709228000	4.058662000	-0.448984000
1	3.797048000	5.648479000	-0.400111000
16	4.776313000	2.201949000	0.044218000
6	5.022292000	3.893696000	-0.101190000
79	1.339611000	-1.343736000	0.057771000
17	2.840684000	-3.086928000	0.378474000
1	6.020415000	4.294220000	-0.041596000
1	-4.264135000	-3.863476000	-0.207417000
1	-2.501955000	3.978650000	-0.272115000
Bis-protonated complex, $AuClLH_2^+$			
6	-2.304197000	-3.353638000	-0.079219000
6	-1.398118000	-2.254292000	-0.109131000
6	-1.919622000	-0.948376000	-0.292310000
6	-3.323828000	-0.773042000	-0.450813000
6	-4.136439000	-1.870554000	-0.405148000
7	-3.598365000	-3.097755000	-0.224917000

1	-5.207395000	-1.828313000	-0.507150000
6	-1.840770000	-4.750395000	0.113854000
1	-1.321188000	-4.841414000	1.068250000
1	-1.126660000	-5.017037000	-0.665359000
1	-2.674002000	-5.447948000	0.090865000
8	-0.148867000	-2.634253000	0.043423000
6	-1.107672000	0.247771000	-0.327592000
1	-1.613025000	1.199110000	-0.436709000
6	-3.962670000	0.572698000	-0.697566000
1	-5.042509000	0.451757000	-0.768570000
1	-3.613285000	0.976415000	-1.653687000
8	-3.632175000	1.457325000	0.366513000
15	-4.383594000	2.946863000	0.419738000
8	-5.833259000	2.756506000	0.108200000
8	-3.879978000	3.577668000	1.676781000
8	-3.722988000	3.688650000	-0.888405000
7	0.170885000	0.248966000	-0.201047000
7	0.924831000	1.391302000	-0.222519000
6	2.260678000	1.262942000	-0.097529000
8	2.759233000	0.087739000	0.053525000
6	3.114194000	2.402128000	-0.133789000
6	2.786261000	3.732715000	-0.340774000
6	3.907991000	4.571987000	-0.316630000
1	1.783691000	4.095266000	-0.511505000
1	3.866063000	5.640109000	-0.458298000
16	4.830654000	2.201818000	0.088838000
6	5.074942000	3.880436000	-0.093543000
79	1.345513000	-1.349752000	0.055776000
17	2.821803000	-3.080543000	0.350669000
1	6.073242000	4.280360000	-0.027776000
1	-4.233764000	-3.888052000	-0.193101000
1	-2.869400000	4.076418000	-0.664452000
1	0.453815000	2.282006000	-0.317437000

Table S8. Calculated IR spectra of different protonated species of complex formed by gold(III) and hydrazone derived from pyridoxal 5′-phosphate and thiophene-2-carbohydrazide (**PLP-T2H**)

Deprotonated complex, $AuClL^-$		Monoprotonated complex, $AuClLH^0$		*Bis*-protonated complex, $AuClLH_2^+$	
Frequency, cm^{-1}	Relative intensity	Frequency, cm^{-1}	Relative intensity	Frequency, cm^{-1}	Relative intensity
18.7723	7.2373	15.2268	2.6552	21.656	21.6829
22.3096	4.164	22.1179	14.9727	26.5272	7.4364

28.2146	9.3348	25.6172	20.3708	29.5737	23.7283
33.1222	1.3514	33.5891	0.8977	31.9483	1.5373
44.9292	2.1428	44.3766	1.4457	51.2087	2.5479
61.1267	0.1879	60.913	2.7296	60.932	3.2589
65.6034	1.8584	63.0752	0.8659	65.4997	0.3264
94.6029	1.9091	93.8449	4.37	92.3317	2.9658
98.5569	4.4778	97.91	4.6496	99.2438	8.383
109.9125	4.2336	108.8064	3.0029	108.1278	1.9113
114.5507	2.1111	115.545	2.2306	117.4014	5.4824
143.3104	9.2056	136.3144	0.2612	141.8767	11.946
153.5427	3.614	141.0036	8.0521	145.8984	10.0704
158.2031	7.8836	153.9934	14.8993	150.3289	2.9238
174.8751	0.7166	173.7183	0.6504	171.9309	0.8378
185.1129	2.7104	181.8639	7.2409	177.3317	7.7963
204.3381	2.3355	204.8321	5.814	202.0479	6.6604
219.6431	3.5757	217.4627	7.9851	210.7691	7.4099
227.6346	8.229	227.9804	7.9813	217.9017	10.9851
239.7053	7.9349	240.6525	6.9031	239.0167	9.3642
259.7037	6.9384	259.0671	6.4752	260.3117	3.8516
284.7818	69.5199	288.5712	57.4023	282.1972	54.8277
324.0684	4.7696	321.3824	22.3118	316.7275	24.5557
325.4502	77.2359	332.6923	61.2027	329.4305	1.0714
339.6194	4.8074	333.7024	1.4735	341.795	7.3451
348.407	6.1227	347.0612	5.1451	352.272	45.669
372.0533	31.4767	368.4721	21.6133	363.0745	12.2445
391.521	17.6126	393.0688	16.0224	391.6387	5.245
400.0054	55.1408	399.9298	77.412	398.1518	81.0328
433.296	0.2318	439.7555	5.5698	412.4335	12.6429
442.7811	1.4954	451.5342	1.4115	438.2165	5.5035
462.5166	130.6811	462.4686	103.7818	461.7545	112.7664
476.4087	39.0644	475.7238	62.0268	466.9731	0.2703
480.8671	2.1346	483.2865	7.9308	476.4638	62.6816
489.8338	56.2821	487.1769	46.5854	485.9274	38.1662
494.0098	38.5527	491.5415	24.6293	490.9301	40.1814
540.6337	83.3731	537.4987	70.9254	517.4927	78.4757
574.698	6.8369	566.1423	0.6018	534.7277	85.6745
578.7625	13.2653	573.8357	3.604	564.1236	4.2431
581.6024	9.7918	579.5059	1.2574	567.2194	18.6385
591.7263	52.8931	593.0089	16.0457	572.2047	29.1245
632.7358	35.9975	632.1995	17.5555	587.276	18.7592

640.9592	42.1431	639.4012	61.1202	628.2378	30.2024
681.8482	70.825	684.1274	54.3627	634.3136	57.341
692.7708	13.8387	697.9128	22.9272	684.2158	48.4156
727.8764	28.2929	731.5422	45.2043	698.0051	45.8518
734.2	101.7711	740.8891	84.4685	726.8022	41.2658
752.9137	12.8949	754.2121	27.7274	751.7814	262.6595
761.6508	89.0171	757.6676	188.6535	755.2998	36.0152
764.3635	52.6803	766.3974	55.4151	760.8559	83.1482
776.0281	305.718	775.2315	18.9396	766.9177	78.8221
787.6874	52.2845	781.0477	290.47	779.1274	18.2591
827.9388	136.8831	794.571	104.1517	786.9214	271.5275
860.2388	80.7243	842.9182	141.2906	800.1542	113.6818
879.7262	5.7703	862.3332	97.2208	846.1646	149.3674
903.9714	1.6306	883.8843	4.4805	869.3673	57.7054
923.8135	43.121	903.6522	1.9153	885.8033	2.1228
950.2498	0.5381	916.459	12.6747	893.4988	23.1061
956.2961	8.1449	943.5362	12.0267	925.0928	7.3282
971.7822	62.3879	954.5617	0.6699	959.385	11.3445
1002.359	43.9119	986.5377	51.3013	961.9882	0.0672
1018.674	243.3885	1011.826	64.973	989.2527	36.6658
1025.313	133.4264	1021.504	156.3397	1018.904	54.7586
1031.264	254.3875	1030.557	145.4395	1023.513	119.7778
1054.101	1.5327	1044.496	416.2629	1045.525	329.1334
1064.148	16.2091	1056.864	8.1754	1053.451	76.1004
1071.911	4.1856	1068.407	18.6915	1057.912	9.3998
1084.906	56.2513	1081.373	17.4497	1075.826	50.952
1093.319	220.2461	1089.181	53.3641	1089.521	38.2542
1105.496	5.4502	1097.055	196.947	1099.181	199.7434
1147.777	262.7999	1106.811	6.8245	1106.234	21.966
1214.495	260.179	1158.232	627.7096	1118.805	246.3554
1249.162	53.4395	1224.99	146.55	1188.535	298.7147
1255.452	30.5738	1249.383	61.5414	1218.56	205.5473
1279.138	443.5736	1254.378	18.4456	1256.342	7.4484
1290.001	232.586	1266.591	239.5269	1263.821	211.8392
1310.488	93.093	1285.769	432.9968	1269.674	14.8012
1327.416	232.5184	1318.331	52.4137	1289.84	411.8434
1331.087	108.1	1330.322	92.5336	1322.669	112.2817
1389.66	37.1045	1343.466	285.4482	1337.416	120.1894
1390.897	131.1602	1388.41	164.7585	1347.351	194.7541
1402.181	64.9457	1391.591	216.3659	1377.484	226.3317

1408.33	35.3355	1403.302	99.3884	1390.055	389.7561
1419.252	129.7943	1415.442	236.3648	1398.853	22.5374
1452.151	232.0559	1427.714	19.2002	1414.988	256.7415
1465.405	20.2111	1436.296	120.8666	1421.04	230.4939
1466.503	10.8045	1447.525	453.3203	1434.628	70.7347
1485.508	3.6648	1451.66	16.8883	1443.81	488.7312
1523.144	8.8694	1476.867	799.8414	1451.614	17.6858
1534.65	955.9657	1500.629	680.2981	1477.165	138.4993
1551.433	118.5564	1523.723	167.7832	1490.085	221.2031
1564.461	110.8523	1530.383	438.6527	1516.765	109.4952
1601.977	65.5642	1557.012	227.0998	1524.159	27.3118
1650.538	168.0157	1614.644	22.9098	1550.318	307.629
3010.609	45.5468	1647.622	22.0256	1596.742	1527.509
3034.735	23.9607	1669.025	66.7679	1619.655	4.0515
3078.809	39.413	3013.925	38.1753	1662.588	42.7761
3080.294	17.6327	3047.941	2.5631	1684.122	17.6787
3133.113	24.7766	3082.571	31.2686	3008.502	35.6805
3164.497	49.8157	3102.264	0.9252	3048.942	1.597
3196.148	35.1557	3136.847	16.6582	3087.873	21.284
3210.953	7.5947	3207.957	55.6071	3103.566	0.202
3226.845	2.2184	3214.025	6.1772	3141.445	13.3566
3247.918	1.8569	3229.116	3.5552	3186.952	131.4107
3812.707	86.0503	3248.284	2.2991	3210.418	0.119
		3248.696	2.5352	3230.466	3.9442
		3546.027	385.9389	3250.009	6.6844
		3811.389	89.5293	3252.997	6.9192
				3535.122	341.5726
				3573.78	157.8683
				3811.157	92.5468

Table S9. Calculated TD-DFT spectra of different protonated species of complex formed by gold(III) and hydrazone derived from pyridoxal 5′-phosphate and thiophene-2-carbohydrazide (**PLP-T2H**)

Deprotonated complex, $AuClL^-$		Monoprotonated complex, $AuClLH^0$		*Bis*-protonated complex, $AuClLH_2^+$	
λ, nm	Oscillator strength	λ, nm	λ, nm	Oscillator strength	λ, nm
479.62	0	445.31	0.0001	454.4	0.0001
372.95	0.0022	388.13	0.5268	375.19	0.0925
370.67	0.2977	349.87	0	366.02	0.0004
317.28	0.0265	327.68	0.0794	361.33	0.2318

310.36	0.0009	324.99	0.0002	317.1	0.0016
308.23	0.5955	315.74	0.3514	308.2	0.6769
290.71	0.0001	292.33	0.0003	302.43	0.0001
289.53	0.0017	284.4	0.0466	297.03	0.0016
280.21	0.0416	277.05	0.0378	286.16	0.0171
266.05	0.3783	273.82	0.0009	285.39	0.0205
258.06	0.072	267.81	0.0003	282.9	0.0038
254.46	0.0002	265.47	0.0032	280.17	0.0293
249.15	0.0161	264.37	0.1025	277.19	0.0004
248.74	0.1223	260.11	0.0897	271.62	0.0542
243.87	0.0025	258.64	0.1115	264.91	0.1057
239.32	0.0173	250.86	0.0013	262.35	0.0254
238.45	0.0023	246.74	0.0005	258.45	0.0585
236.47	0.0152	244.01	0.2354	252.8	0.1127
231.82	0.0194	237.97	0.0001	245.29	0.1069
228.49	0.0523	231.92	0.0651	243.37	0.0262
225.58	0.1046	231.47	0.0291	240.76	0.0001
223.55	0.0066	230.69	0.0012	236.02	0.0009
220.62	0.0037	230.04	0.0433	229.63	0.0016
219.71	0.0045	225.23	0.0026	227.31	0.0374
217.52	0.0431	224.53	0.001	225.41	0.0238
214.78	0.0042	221.34	0.0329	222.41	0.0012
211.49	0.0809	218.05	0.0168	222.16	0.0069
209.76	0.0002	217.6	0.0076	219.49	0.0624
208.04	0.2373	214.64	0.095	216.91	0.0024
206.29	0.0088	208.29	0.0072	213.91	0.0742

Table S10. Optimized geometry (*xyz*-coordinates) of different protonated species of complex formed by gold(III) and hydrazone derived from pyridoxal 5′-phosphate and thiophene-3-carbohydrazide (**PLP-T3H**)

Deprotonated complex, $AuClL^{-}$			
6	-2.795318000	-3.094306000	-0.085275000
6	-1.731069000	-2.136235000	-0.113977000
6	-2.061175000	-0.772306000	-0.297191000
6	-3.435438000	-0.433183000	-0.456336000
6	-4.365471000	-1.442489000	-0.413979000
7	-4.054514000	-2.739107000	-0.232481000
1	-5.417717000	-1.214944000	-0.530220000
6	-2.473823000	-4.544209000	0.118548000
1	-1.946977000	-4.700555000	1.061511000
1	-1.818640000	-4.916351000	-0.671035000
1	-3.395004000	-5.120792000	0.121777000
8	-0.526506000	-2.659683000	0.035296000
6	-1.102665000	0.297346000	-0.333422000
1	-1.455241000	1.311271000	-0.467291000
6	-3.897125000	0.981414000	-0.682872000
1	-4.965816000	0.983663000	-0.894246000
1	-3.392564000	1.417034000	-1.550347000
8	-3.631477000	1.768150000	0.485333000
15	-3.804548000	3.409876000	0.383978000
8	-5.021787000	3.728925000	-0.426798000
8	-3.589752000	3.918584000	1.774756000
8	-2.507327000	3.816406000	-0.552508000
7	0.176217000	0.174292000	-0.203309000
7	1.004333000	1.266658000	-0.248963000
6	2.258290000	0.935660000	-0.105369000
8	2.681971000	-0.304478000	0.061608000
6	3.280837000	1.978690000	-0.126769000
6	3.011813000	3.373244000	-0.304918000
6	4.140454000	4.127693000	-0.285060000
1	2.020499000	3.775041000	-0.440166000
1	4.233496000	5.194621000	-0.394399000
6	4.620331000	1.718434000	0.024070000
1	5.091932000	0.763051000	0.172608000
16	5.552234000	3.153698000	-0.048777000
79	1.118129000	-1.555937000	0.058623000
17	2.403457000	-3.478766000	0.360331000
1	-1.733484000	3.964075000	0.002773000
Monoprotonated complex, $AuClLH^{0}$			
6	-2.841404000	-3.017785000	-0.073787000

6	-1.773554000	-2.080279000	-0.109278000
6	-2.078022000	-0.706021000	-0.293854000
6	-3.437464000	-0.315607000	-0.452877000
6	-4.416092000	-1.267911000	-0.410398000
7	-4.083345000	-2.564933000	-0.225315000
1	-5.465919000	-1.052666000	-0.515588000
6	-2.603594000	-4.470816000	0.125746000
1	-2.072811000	-4.635813000	1.063562000
1	-1.968546000	-4.857952000	-0.671526000
1	-3.537595000	-5.027059000	0.140724000
8	-0.593914000	-2.636100000	0.037661000
6	-1.089100000	0.344028000	-0.335675000
1	-1.415635000	1.365857000	-0.469648000
6	-3.849742000	1.117957000	-0.677587000
1	-4.919503000	1.160878000	-0.877206000
1	-3.338510000	1.518983000	-1.557636000
8	-3.531527000	1.879012000	0.484085000
15	-3.638381000	3.534840000	0.386801000
8	-4.844938000	3.890806000	-0.422194000
8	-3.400151000	4.025504000	1.778368000
8	-2.327412000	3.878564000	-0.551711000
7	0.184562000	0.180772000	-0.206623000
7	1.036543000	1.241101000	-0.253448000
6	2.286882000	0.875372000	-0.108829000
8	2.671539000	-0.375060000	0.058120000
6	3.333212000	1.887436000	-0.130018000
6	3.100361000	3.288743000	-0.308986000
6	4.248932000	4.011277000	-0.287542000
1	2.120393000	3.716793000	-0.445608000
1	4.371850000	5.075131000	-0.396741000
6	4.666652000	1.590795000	0.023001000
1	5.112056000	0.623018000	0.172784000
16	5.633699000	2.998674000	-0.048850000
79	1.083368000	-1.577326000	0.058000000
17	2.312705000	-3.523636000	0.359614000
1	-1.552057000	4.023539000	0.002486000
1	-4.834729000	-3.245066000	-0.197892000
Bis-protonated complex, $AuClLH_2^+$			
6	-2.852791000	-3.014173000	-0.053375000
6	-1.788226000	-2.066077000	-0.096145000
6	-2.107290000	-0.695414000	-0.267517000
6	-3.469113000	-0.309083000	-0.416395000
6	-4.439309000	-1.268642000	-0.369660000
7	-4.093799000	-2.564119000	-0.191827000

1	-5.491655000	-1.062191000	-0.465444000
6	-2.603452000	-4.464913000	0.138752000
1	-2.054568000	-4.627264000	1.066530000
1	-1.980435000	-4.845546000	-0.671209000
1	-3.534113000	-5.025490000	0.169234000
8	-0.609529000	-2.630996000	0.034512000
6	-1.126025000	0.365899000	-0.306008000
1	-1.476388000	1.384923000	-0.421478000
6	-3.887740000	1.122325000	-0.644277000
1	-4.965647000	1.166595000	-0.789448000
1	-3.421753000	1.500320000	-1.559797000
8	-3.503030000	1.902132000	0.482940000
15	-3.639031000	3.558858000	0.361567000
8	-4.871607000	3.870892000	-0.424449000
8	-3.371776000	4.081188000	1.735181000
8	-2.348572000	3.891543000	-0.610103000
7	0.138432000	0.170721000	-0.186770000
7	1.060846000	1.183537000	-0.222792000
6	2.358676000	0.855478000	-0.094176000
8	2.674560000	-0.379489000	0.048609000
6	3.379953000	1.867186000	-0.117725000
6	3.182062000	3.276950000	-0.291525000
6	4.350975000	3.961659000	-0.264081000
1	2.229747000	3.763652000	-0.433504000
1	4.503163000	5.021867000	-0.369314000
6	4.712378000	1.534906000	0.036537000
1	5.128298000	0.553402000	0.183311000
16	5.705887000	2.908641000	-0.026818000
79	1.061935000	-1.586654000	0.051079000
17	2.259722000	-3.522803000	0.315945000
1	-1.603949000	4.185435000	-0.073238000
1	-4.842365000	-3.248727000	-0.160338000
1	0.733507000	2.135491000	-0.326217000

Table S11. Calculated IR spectra of different protonated species of complex formed by gold(III) and hydrazone derived from pyridoxal 5′-phosphate and thiophene-3-carbohydrazide (**PLP-T3H**)

Deprotonated complex, $AuClL^-$		Monoprotonated complex, $AuClLH^0$		*Bis*-protonated complex, $AuClLH_2^+$	
Frequency, cm^{-1}	Relative intensity	Frequency, cm^{-1}	Relative intensity	Frequency, cm^{-1}	Relative intensity
16.4947	10.8957	18.0541	13.2876	21.6168	10.2486

22.7206	5.1545	20.4027	15.1439	31.9349	11.3574
33.7063	2.5194	32.4195	1.8585	36.5659	11.922
38.7305	3.4295	37.0306	6.66	38.6688	16.7972
47.3244	2.7991	47.2895	0.4983	57.6717	2.6793
58.8283	0.3384	59.4687	0.1732	63.7725	1.9356
60.6285	0.8853	63.4899	1.6561	69.9397	1.9792
81.6162	0.2577	79.1615	1.9282	77.0419	1.6173
99.4964	4.2052	99.0939	4.646	100.7038	7.0186
109.3594	3.3646	109.3692	3.0927	111.3412	2.3625
116.297	2.3705	117.1446	1.8843	120.1322	3.7646
137.7613	8.2833	133.6587	2.4079	135.439	1.3748
153.136	1.4424	146.8242	13.4943	151.1797	17.6636
160.6423	10.3044	163.5223	8.3351	161.9574	5.4149
182.4208	1.5379	182.0973	8.792	179.2632	10.508
184.6263	2.4748	183.9569	0.6471	189.1321	3.4897
202.8201	2.2306	204.3005	5.3359	198.1945	7.942
217.093	4.935	217.2995	8.8499	212.6837	13.558
224.9359	7.4229	224.2215	9.3973	219.5478	6.6856
238.7373	4.5099	240.2811	3.1731	239.7244	8.0497
263.1304	7.6031	259.8218	7.5239	252.892	6.1916
294.1317	54.3936	300.04	33.9675	309.0792	5.3897
325.8449	87.4849	329.9927	45.4889	335.4126	2.2847
328.1379	3.9001	334.3575	45.9756	337.3479	18.9456
340.1137	4.9829	338.6187	3.6371	343.4474	7.4087
349.0414	5.4057	347.9954	4.6103	357.5784	45.0653
374.7052	39.2825	374.3105	30.1116	372.2904	5.1099
399.4163	58.7978	402.8115	85.423	410.1632	10.4109
411.302	17.1835	412.5692	16.0941	417.7307	7.0363
442.1767	2.5471	442.4728	8.424	442.1509	7.602
443.4217	0.4373	460.2589	4.977	453.3685	157.1034
463.3227	110.3687	463.6326	77.4278	464.659	87.2404
468.669	0.6858	471.5512	4.2679	466.8018	0.3921
476.3635	41.2208	475.2689	58.4481	477.3427	12.6813
489.3809	66.3429	488.3137	64.9857	486.1416	93.69
492.7711	38.7036	491.273	24.1902	492.5128	45.3444
540.8086	85.2276	538.0021	70.4928	509.836	98.572
578.8739	18.0318	570.4356	0.907	536.4147	77.4286
582.2441	4.9982	581.1256	0.4587	568.9673	8.354
599.7126	48.3252	600.7424	21.9131	577.8552	1.9227
604.9159	6.1494	604.4041	3.8423	595.4411	14.6871

636.4684	61.8006	636.9573	54.5428	597.6973	32.8417
642.4885	32.5369	642.9706	22.2075	635.4792	72.4219
657.4711	22.4859	662.7147	17.0162	642.0771	8.5123
683.4059	25.854	684.3575	21.2591	653.9433	28.766
713.4619	28.8923	716.1275	33.14	687.1226	3.4325
736.1716	73.6688	739.7896	71.4189	717.6635	50.0121
757.1466	4.3272	757.0081	139.0507	737.5633	42.1576
761.6901	125.7591	762.476	99.8304	753.4343	223.3478
772.7477	319.6224	775.0043	305.3856	764.1533	140.2278
787.8056	37.2168	779.8205	1.346	772.7189	242.7848
801.8323	11.1005	800.1554	33.2351	782.2479	2.2164
826.7268	120.1105	806.9972	81.4041	799.434	37.9297
836.6684	45.366	839.8779	36.7044	812.101	73.7405
862.6738	164.7995	851.9694	134.4168	847.2492	41.5423
894.7292	6.9856	865.4858	205.7076	859.4625	134.0986
922.8302	41.2422	896.5333	10.2462	871.3831	98.2594
926.0763	0.6123	926.3199	6.5287	906.7526	73.3628
959.3748	7.6146	927.5198	0.6561	916.6957	0.0156
961.1299	31.3781	947.2744	9.5147	939.7958	6.558
980.7359	43.3824	966.5965	8.0552	956.5633	7.3134
1008.573	126.7044	990.7313	50.7466	969.667	76.4691
1023.084	164.0626	1017.884	55.4511	995.7283	17.9231
1030.151	41.994	1023.334	162.4927	1017.395	47.3518
1033.68	370.7447	1038.922	207.0863	1024.154	183.1319
1054.153	1.4153	1048.638	434.3614	1048.587	337.8167
1071.088	11.2361	1057.172	10.7119	1058.768	9.3522
1089.698	188.7867	1081.522	23.8722	1062.034	99.9919
1093.621	94.557	1091.758	178.9019	1080.985	41.0412
1103.035	1.7204	1096.598	120.2176	1096.208	257.9995
1143.464	122.8972	1105.79	0.9955	1127.032	1.5201
1212.03	156.7565	1151.655	251.0319	1139.492	45.2193
1225.759	97.4345	1224.499	42.3521	1184.14	91.7589
1256.632	25.3357	1230.207	131.4526	1219.237	211.2307
1280.029	446.5726	1257.205	5.018	1256.6	14.4853
1290.679	213.0915	1275.241	161.0767	1258.101	8.3871
1309.829	168.9186	1286.462	434.2131	1274.128	175.7876
1325.541	169.0427	1317.977	250.9862	1291.052	428.3984
1331.824	104.9686	1335.681	28.4155	1330.417	173.3815
1391.533	133.4505	1342.835	297.9239	1335.617	1.5159
1398.664	10.7555	1390.947	185.0241	1346.84	536.0094

1407.519	21.0804	1402.169	9.769	1386.417	179.0263
1410.727	3.0224	1408.635	88.0539	1396.273	135.8163
1426.174	52.0956	1422.88	133.7056	1404.916	20.8823
1459.061	129.023	1427.503	15.2951	1422.044	256.069
1465.303	25.9271	1438.956	59.6195	1422.817	18.7787
1465.889	10.8081	1452.536	16.1034	1437.691	84.5781
1486.249	2.1721	1459.893	134.1511	1451.9	18.8231
1519.669	9.1488	1483.886	521.0872	1457.543	467.7288
1531.785	560.7618	1506.109	617.6673	1484.347	77.0489
1553.988	25.95	1522.439	62.3814	1503.961	206.5779
1569.234	522.6784	1534.597	500.8449	1523.245	86.2023
1603.1	85.2017	1555.399	538.3338	1534.072	440.8246
1651.506	186.3604	1619.526	48.2046	1550.392	95.7963
3013.855	41.7508	1652.969	37.7843	1610.02	1197.97
3034.767	23.1342	1671.816	70.9662	1624.009	8.3717
3078.303	45.0594	3018.391	32.711	1666.648	46.4664
3080.242	17.6644	3048.65	2.3267	1685.199	25.2936
3133.891	23.3737	3083.731	37.8922	3011.155	27.9467
3164.219	47.4707	3102.605	0.8952	3049.65	1.6083
3189.827	46.6754	3137.969	16.0769	3091.426	30.9725
3226.7	2.2065	3197.947	73.957	3103.965	0.181
3253.381	0.7367	3228.484	3.2399	3142.367	12.7355
3261.4	7.482	3253.624	3.5829	3167.214	135.424
3812.074	87.7197	3254.737	1.5231	3217.771	0.6954
		3260.945	8.922	3257.759	8.1479
		3553.651	382.8766	3259.412	11.648
		3810.254	92.4685	3262.75	15.2328
				3541.838	350.1686
				3588.832	175.6689
				3814.997	104.5049

Table S12. Calculated TD-DFT spectra of different protonated species of complex formed by gold(III) and hydrazone derived from pyridoxal 5′-phosphate and thiophene-3-carbohydrazide (**PLP-T3H**)

Deprotonated complex, $AuClL^-$		Monoprotonated complex, $AuClLH^0$		*Bis*-protonated complex, $AuClLH_2^+$	
λ, nm	Oscillator strength	λ, nm	λ, nm	Oscillator strength	λ, nm
469.28	0.0001	433.21	0.0001	448.18	0.0001
369.97	0.0011	378.94	0.4116	374.1	0.0663
366.18	0.2188	349.31	0	364	0.0003

315.91	0.026	326.01	0.0576	359.5	0.1836
305.81	0.0001	319.76	0.0002	312.03	0.0001
300.15	0.5659	309.81	0.4295	301.88	0
290.03	0.0014	292.28	0.0003	298.89	0.3441
288.32	0.0011	284.42	0.0361	295.11	0.0968
279.28	0.0348	281.84	0.0163	288.38	0.0156
261.96	0.4275	273.62	0.0043	284.65	0.0518
257.03	0.0004	268.4	0.0001	281.47	0.2212
253.73	0.0026	264.92	0.0001	280.51	0.0032
245.28	0.0681	261.76	0.0506	278.21	0.0002
244.42	0.219	259.62	0.1216	269.96	0.0615
243.27	0.0211	255.14	0.1667	263.39	0.1164
238.92	0.0332	250.09	0.0032	260.91	0.1096
236.56	0.0007	245.33	0.0001	257.54	0.0194
233.48	0.0251	238.74	0.0001	250.24	0.1415
228.79	0.0095	232.72	0.2547	242.99	0.0007
226.25	0.0746	231.38	0.0209	238.99	0.0002
222.48	0.0032	230.65	0.0433	238.02	0.0011
220.62	0.019	229.45	0.0046	236.62	0.0942
218.96	0.0039	225.19	0.1071	231.18	0.0029
218.33	0.0084	224.74	0.0114	228.5	0.026
215.1	0.0056	223.1	0.0034	224.39	0.0632
212.97	0.1672	220.26	0.0681	222.64	0.0182
210.58	0.1959	218.49	0.0093	220.52	0.0043
209	0.0005	216.73	0.0542	218.65	0.017
206.77	0.1986	213.98	0.1693	217.47	0.1826
206.17	0.0299	207.94	0.2038	210.97	0.0203

Table S13. Optimized geometry (*xyz*-coordinates) of different protonated species of complex formed by gold(III) and hydrazone derived from pyridoxal 5′-phosphate and isoniazid (**PLP-INH**)

Deprotonated complex, $AuClL^-$			
6	-2.377656000	-3.341897000	-0.099910000
6	-1.417881000	-2.278036000	-0.121941000
6	-1.887082000	-0.954935000	-0.306879000
6	-3.288942000	-0.758960000	-0.471754000
6	-4.108627000	-1.859385000	-0.433555000
7	-3.665141000	-3.117368000	-0.252428000
1	-5.178252000	-1.742835000	-0.553925000

6	-1.909079000	-4.751112000	0.102995000
1	-1.371557000	-4.853817000	1.047235000
1	-1.216983000	-5.052283000	-0.685361000
1	-2.766088000	-5.419341000	0.102656000
8	-0.167765000	-2.675725000	0.035213000
6	-1.044728000	0.206698000	-0.337734000
1	-1.501883000	1.178615000	-0.462960000
6	-3.896361000	0.598415000	-0.706144000
1	-4.964115000	0.487730000	-0.891597000
1	-3.456937000	1.070554000	-1.590566000
8	-3.684823000	1.428601000	0.441816000
15	-4.255459000	2.982321000	0.411958000
8	-5.623836000	2.994188000	-0.194923000
8	-3.959472000	3.533079000	1.771475000
8	-3.258393000	3.682325000	-0.697903000
7	0.240156000	0.213807000	-0.207447000
7	0.952675000	1.385850000	-0.248966000
6	2.229199000	1.183630000	-0.102549000
8	2.780358000	-0.001210000	0.063868000
79	1.353877000	-1.410479000	0.059754000
17	2.829105000	-3.187461000	0.377804000
1	-2.436996000	3.963879000	-0.279765000
6	3.151804000	2.339827000	-0.119748000
6	2.682288000	3.641441000	-0.298305000
6	3.597768000	4.682499000	-0.305150000
7	4.914718000	4.512843000	-0.149330000
6	5.354190000	3.264165000	0.021017000
6	4.522793000	2.152277000	0.043430000
1	1.629219000	3.836538000	-0.428762000
1	3.255555000	5.701125000	-0.442452000
1	6.423212000	3.140965000	0.146284000
1	4.934126000	1.164992000	0.184830000
Monoprotonated complex, $AuClLH^0$			
6	-2.521730000	-3.219740000	-0.080877000
6	-1.532878000	-2.197440000	-0.113461000
6	-1.948662000	-0.853187000	-0.300968000
6	-3.334549000	-0.574469000	-0.468039000
6	-4.231625000	-1.603936000	-0.431076000
7	-3.794048000	-2.869083000	-0.240865000
1	-5.294663000	-1.477080000	-0.545233000
6	-2.170027000	-4.647985000	0.125096000
1	-1.633641000	-4.767448000	1.066605000
1	-1.501922000	-4.986430000	-0.667277000
1	-3.058198000	-5.274847000	0.136709000
8	-0.313950000	-2.657518000	0.038540000

	6	-1.048613000	0.274474000	-0.335776000
	1	-1.458119000	1.266549000	-0.464388000
	6	-3.860966000	0.820960000	-0.694919000
	1	-4.930639000	0.778282000	-0.894692000
	1	-3.382755000	1.261838000	-1.574767000
	8	-3.603762000	1.605042000	0.465978000
	15	-3.947900000	3.231137000	0.402865000
	8	-5.223763000	3.419689000	-0.354298000
	8	-3.728010000	3.729685000	1.794589000
	8	-2.739803000	3.772501000	-0.576953000
	7	0.232618000	0.212454000	-0.204172000
	7	0.998631000	1.340304000	-0.246254000
	6	2.268064000	1.073213000	-0.102225000
	8	2.755064000	-0.136513000	0.062347000
	79	1.270616000	-1.467672000	0.058283000
	17	2.652973000	-3.306901000	0.359892000
	6	3.246324000	2.179814000	-0.121572000
	6	2.839233000	3.500428000	-0.312909000
	6	3.804745000	4.495598000	-0.322402000
	7	5.110215000	4.261873000	-0.157279000
	6	5.489172000	2.995163000	0.025484000
	6	4.605313000	1.924878000	0.051309000
	1	1.797603000	3.744816000	-0.451455000
	1	3.513809000	5.528492000	-0.469776000
	1	6.550192000	2.822192000	0.158113000
	1	4.967551000	0.920024000	0.202749000
	1	-1.962784000	3.999338000	-0.053522000
	1	-4.488084000	-3.607930000	-0.216213000
	Bis-protonated complex, $AuClLH_2^+$			
	6	-2.380719000	-3.301505000	-0.024231000
	6	-1.431923000	-2.238518000	-0.097101000
	6	-1.907283000	-0.916399000	-0.292211000
	6	-3.305882000	-0.690451000	-0.442926000
	6	-4.157833000	-1.755153000	-0.367150000
	7	-3.664326000	-2.997817000	-0.162153000
	1	-5.227368000	-1.675576000	-0.461059000
	6	-1.969887000	-4.709512000	0.201086000
	1	-1.417265000	-4.789601000	1.137582000
	1	-1.299103000	-5.034253000	-0.594631000
	1	-2.832500000	-5.369907000	0.234883000
	8	-0.196466000	-2.666395000	0.029120000
	6	-1.054555000	0.249171000	-0.336105000
	1	-1.528366000	1.215626000	-0.456417000
	6	-3.895627000	0.672810000	-0.714609000

	1 -4.980305000 0.592599000 -0.765540000
	1 -3.546113000 1.036328000 -1.686761000
	8 -3.513307000 1.571661000 0.319474000
	15 -4.211223000 3.088991000 0.345422000
	8 -5.672208000 2.939610000 0.066894000
	8 -3.660788000 3.734761000 1.574739000
	8 -3.551016000 3.769689000 -0.995142000
	7 0.221463000 0.203765000 -0.192378000
	7 1.021683000 1.320583000 -0.225667000
	6 2.336770000 1.141097000 -0.068349000
	8 2.800793000 -0.035968000 0.077045000
	79 1.340566000 -1.436234000 0.060205000
	17 2.749693000 -3.220956000 0.348976000
	6 3.254066000 2.286224000 -0.074294000
	6 2.844983000 3.571008000 0.282403000
	6 3.789041000 4.588081000 0.247921000
	7 5.060229000 4.399719000 -0.109945000
	6 5.440774000 3.164456000 -0.442695000
	6 4.583755000 2.073531000 -0.436451000
	1 1.840702000 3.790956000 0.613475000
	1 3.505157000 5.595077000 0.525125000
	1 6.476833000 3.035806000 -0.728305000
	1 4.936389000 1.093458000 -0.717831000
	1 -2.681149000 4.135443000 -0.798282000
	1 -4.330087000 -3.761564000 -0.104799000
	1 0.598519000 2.219823000 -0.425314000

Table S14. Calculated IR spectra of different protonated species of complex formed by gold(III) and hydrazone derived from pyridoxal 5′-phosphate and isoniazid (**PLP-INH**)

Deprotonated complex, $AuClL^-$		Monoprotonated complex, $AuClLH^0$		*Bis*-protonated complex, $AuClLH_2^+$	
Frequency, cm^{-1}	Relative intensity	Frequency, cm^{-1}	Relative intensity	Frequency, cm^{-1}	Relative intensity
21.7317	4.1636	16.9233	10.0684	19.9555	8.5799
24.151	4.5597	20.2664	10.439	22.8722	11.2877
26.4231	2.3314	24.6311	0.0288	29.0002	21.6457
30.5269	8.3846	29.2404	12.2648	30.8936	2.383
43.4782	5.9207	43.9502	2.9297	45.5139	8.6028
64.0234	0.4343	60.9527	1.7793	58.1862	1.6874
65.3064	2.1971	62.8494	0.1774	64.8509	4.0045
92.081	0.7769	86.3897	6.0518	88.42	6.7829
100.4952	5.1063	99.7245	5.5193	98.9579	8.9663
108.7465	2.0994	106.9285	2.255	108.1792	2.2574

114.8098	3.5061	115.9207	1.7309	115.1939	7.7603
143.6088	9.1804	135.1844	1.9121	133.3311	1.3377
153.4393	2.9323	141.53	7.9123	143.8545	21.1129
159.6377	5.6305	158.838	15.1906	147.5545	4.0108
179.7605	0.4261	177.4115	2.8296	169.3067	4.3985
185.8078	0.1342	186.216	0.531	185.0072	5.1055
208.9114	3.2575	208.1617	6.0026	205.4232	5.8669
219.9374	5.9606	219.2776	7.722	211.3075	13.3504
228.2558	3.6644	228.1179	4.8004	223.3588	3.0151
238.1336	5.5365	238.3356	5.3502	237.1122	6.665
255.6392	17.8013	252.3541	19.2028	268.0278	13.7376
288.4134	63.7724	295.3436	42.7341	284.5442	48.3884
324.8642	45.2714	326.9131	40.2959	318.1699	31.5223
327.0511	37.7738	333.8417	30.9253	328.9353	1.6236
339.57	3.8838	335.9514	19.6268	343.706	13.0134
348.8123	5.4722	347.2158	4.2156	352.9003	31.1693
373.9576	27.6405	372.988	30.7138	365.7036	13.0579
389.9952	0.1319	388.0956	0.1471	381.1217	0.5229
402.0121	61.9427	399.6784	77.7173	390.4241	17.687
414.6828	34.0383	416.3292	30.6973	398.5026	87.2114
429.0423	0.2926	439.4431	5.6778	420.6595	5.0138
441.8044	1.6606	446.9814	0.961	436.6311	5.9255
462.979	104.6075	463.0226	75.3853	457.7195	97.0075
477.9313	43.8237	475.209	63.2378	475.576	49.9485
488.4748	49.9667	486.5215	61.0525	486.6929	27.3244
492.679	38.008	489.6089	21.5423	488.0588	56.1119
507.7307	9.8547	510.8274	11.1914	495.0473	21.3996
542.2386	85.7747	537.3765	70.484	533.429	57.2914
578.9621	19.2819	568.9057	1.3296	551.6306	121.2405
580.9245	7.7846	578.5562	0.8154	565.4078	19.8935
592.3396	48.627	592.4383	18.5128	571.4177	26.3318
635.7801	79.1882	636.6959	71.9152	585.5947	16.7873
653.9039	9.5793	656.6235	5.6735	631.6316	89.3031
681.1318	4.1923	680.6482	3.8917	642.5736	13.9594
685.3318	20.2216	686.063	18.7584	677.0613	3.3665
710.5944	45.5285	713.3599	47.4345	688.0704	9.4135
724.3467	120.0771	726.794	119.9347	718.5272	65.6073
758.5392	0.9054	756.9779	145.0254	722.6739	56.1435
762.6143	137.8816	762.7882	87.5211	751.7705	271.5828
768.6597	10.3221	767.3278	11.9535	762.9658	16.6057

777.2817	301.4537	777.9547	268.8079	764.8685	39.1457
788.7056	59.1562	779.8075	40.3531	781.3591	23.9113
827.9208	133.923	806.5896	93.9281	787.9398	273.0515
868.774	27.834	853.7786	135.8098	805.3858	100.9143
897.8694	0.0005	868.9454	27.4319	854.1741	135.5479
923.9861	42.5027	897.0253	0.0042	858.5477	25.334
943.5998	0.4591	923.0938	6.8499	886.986	4.9942
963.6422	7.9934	945.3071	6.8508	930.6683	6.2795
976.4827	78.6899	953.9818	8.3952	943.6302	2.1685
1006.239	0.8105	988.1064	49.8148	970.3436	8.4945
1008.604	87.4078	1007.282	0.8658	993.8919	33.2097
1013.237	10.5365	1012.712	5.2556	1005.133	1.275
1020.817	0.2163	1016.619	37.6102	1012.598	0.9847
1022.149	145.6929	1021.579	57.2369	1021.184	86.7475
1029.587	14.9919	1021.724	110.1304	1023.063	1.3543
1032.65	386.3636	1037.061	97.0902	1023.734	118.3088
1054.041	1.3401	1047.378	429.3363	1045.9	304.3077
1070.81	13.8991	1057.083	9.2506	1058.601	11.4019
1089.517	32.8105	1079.988	23.6669	1062.211	131.753
1092.293	227.0156	1090.416	26.5185	1079.261	40.0195
1099.709	14.1836	1094.54	231.943	1095.524	31.5546
1113.209	0.4423	1101.189	29.0755	1099.561	201.5141
1163.297	39.2961	1114.226	0.1026	1125.051	3.0796
1213.03	249.031	1171.01	76.5718	1138.884	14.4861
1240.57	6.6371	1222.987	154.3431	1184.884	14.9999
1255.719	22.0644	1240.768	7.003	1215.979	195.4797
1272.093	10.7158	1254.432	8.7833	1248.346	1.5462
1279.553	448.8412	1264.255	136.5581	1254.226	5.4467
1291.125	175.2041	1272.268	40.4488	1262.113	147.1613
1313.89	56.4506	1287.149	438.4996	1268.795	43.6743
1329.401	125.1594	1324.216	14.9781	1290.424	414.7318
1334.769	186.1058	1331.579	105.0404	1320.458	121.0254
1361.026	39.1172	1349.079	230.1184	1328.482	152.1387
1392.101	125.9848	1363.029	153.1017	1340.195	179.4169
1399.327	51.8902	1391.011	139.6795	1365.081	20.9392
1408.457	20.3983	1403.128	70.7787	1389.245	118.553
1418.229	47.9857	1411.757	56.2789	1398.587	14.1771
1442.346	32.8958	1428.096	1.8851	1417.802	105.9006
1465.25	25.7893	1436.254	102.1998	1418.287	256.3211
1466.524	10.9519	1441.258	48.9676	1433.978	68.0198

1485.167	0.8268	1451.592	16.9986	1443.228	40.4123
1522.199	7.0346	1483.766	96.8062	1449.704	18.2467
1528.52	88.3558	1512.629	187.3808	1482.069	49.516
1548.876	198.4517	1523.329	53.6627	1504.029	97.3546
1556.652	381.5032	1531.025	45.7317	1519.62	173.7162
1601.731	32.784	1538.431	924.3719	1523.828	79.215
1607.62	78.1288	1601.822	20.0552	1534.192	277.8583
1633.91	154.8819	1616.868	59.2887	1588.409	731.1275
1653.272	98.9005	1635.015	57.6208	1601.997	55.8509
3008.313	46.5519	1654.54	17.1529	1619.693	6.968
3035.248	22.3347	1671.763	68.8053	1630.502	1.0464
3078.151	36.1018	3015.083	34.5845	1664.718	71.2084
3080.894	17.1291	3048.887	1.7945	1683.869	47.1042
3134.457	23.533	3083.758	34.5587	3006.506	33.977
3159.204	49.7863	3102.843	0.7055	3049.655	1.6928
3163.175	24.9293	3139.213	14.8035	3087.297	20.925
3165.769	45.7914	3161.76	45.563	3104.116	0.0992
3198.147	32.8494	3165.677	22.3967	3142.736	11.6723
3219.683	2.7235	3201.64	60.3723	3174.544	27.1534
3220.156	3.6694	3220.916	2.3357	3178.004	9.0853
3813.024	86.3964	3221.267	4.3863	3181.404	146.6509
		3249.983	3.4488	3206.143	2.5248
		3544.222	355.1248	3222.71	2.9272
		3811.116	93.3972	3251.809	7.8233
				3537.21	325.9484
				3547.649	196.1639
				3810.4	95.5826

Table S15. Calculated TD-DFT spectra of different protonated species of complex formed by gold(III) and hydrazone derived from pyridoxal 5′-phosphate and isoniazid (**PLP-INH**)

Deprotonated complex, $AuClL^-$		Monoprotonated complex, $AuClLH^0$		*Bis*-protonated complex, $AuClLH_2^+$	
λ, nm	Oscillator strength	λ, nm	Oscillator strength	λ, nm	Oscillator strength
463.32	0.0001	421.51	0.0001	450.81	0.0002
369.19	0.0057	371.42	0.2778	379.43	0.0179
367.67	0.1659	348.22	0	368.26	0.0001
318.68	0.0223	328.81	0.0468	361.58	0.1556
305.92	0.0001	316.97	0.0001	304.37	0.0016
298.85	0.4482	304.51	0.3935	300.76	0.0002
293.49	0.0028	283.12	0.0636	296.18	0.0086
279.69	0.031	277.82	0.0065	292.18	0.0605
266.15	0.0027	276.47	0.0017	289.72	0.0038
263.37	0.0087	268.08	0.0018	287.8	0.0306
261.78	0.4088	267.26	0.047	281.84	0.0128
252.93	0.0934	264.24	0.0155	280.3	0.1657
246.42	0.0097	259.38	0.223	271.96	0.103
243.04	0.1801	254.53	0.0053	270.63	0.0561
241.6	0.1174	252.69	0.0003	268.36	0.0346
239.86	0.0147	248.61	0.0002	265.4	0.0313
239.48	0.0008	247.97	0.0002	258.94	0.3479
234.07	0.0526	240.39	0.0003	253.98	0.1027
231.23	0.0707	237.54	0.4946	249.78	0.0035
229.05	0.0044	233.35	0.0593	247.79	0.0171
225.46	0.0515	232.19	0.0041	240.31	0.014
224.3	0.0029	231.49	0.0091	236.69	0.2966
223.17	0.0018	230.3	0.0025	233.8	0.0449
221.2	0.024	229.87	0.0295	230.04	0.0082
218.67	0.0021	227.85	0.0007	229.62	0.0326
218.19	0.0133	221.37	0.0068	226.14	0.016
217.68	0	220.11	0.0344	225.98	0.0013
217.21	0.003	219.45	0.0011	225.43	0.0175
212.45	0.1902	219.27	0.0004	222.77	0.0792
210.17	0.0637	218.24	0.0646	222.38	0.0282

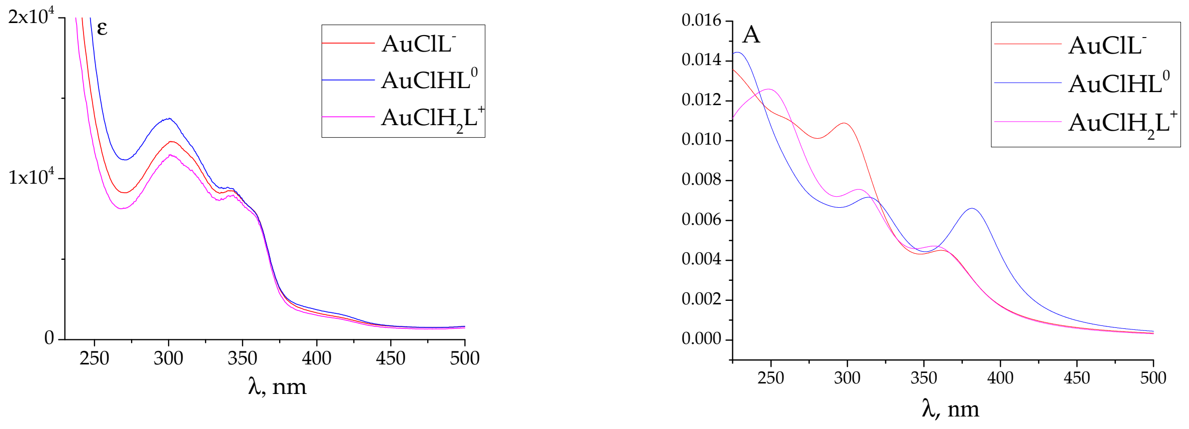

L = PLP-F3H

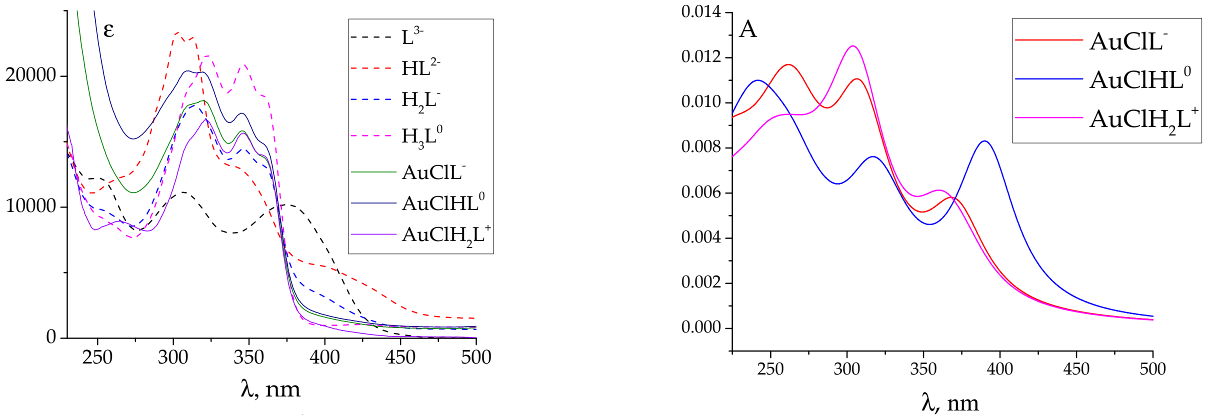

L = PLP-F2H

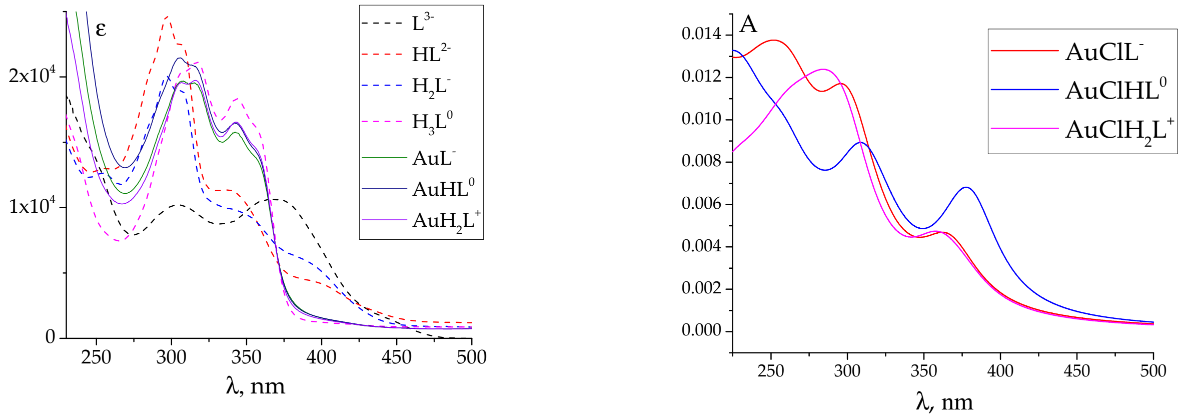

L = PLP-T3H

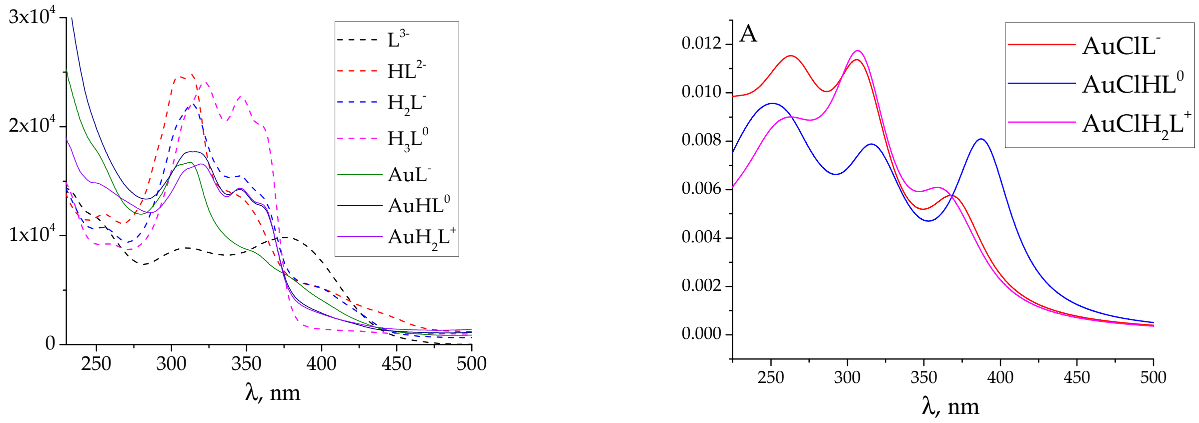

L = PLP-T2H

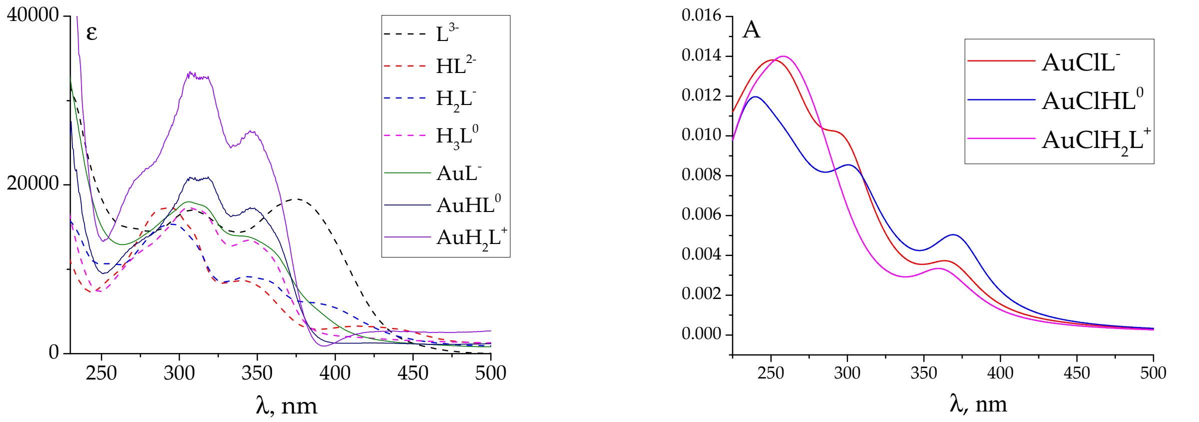

L = PLP-INH

(**a**) (**b**)

Figure S1. (**a**) The observed UV–Vis absorption spectra for protonated forms of gold(III) hydrazone complexes complex (taken from Ref. [30] of main text); (**b**) The simulated UV–Vis absorption spectra for protonated forms of gold(III) hydrazone complexes in the range of 225-500 nm. The theoretical individual bands were described by Lorentz curves with a half-width of 47 nm.

AuCl(PLP-F3H)						
AuClL$^-$	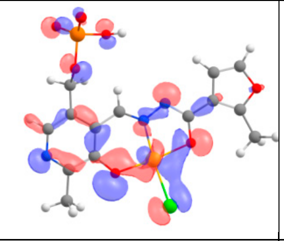	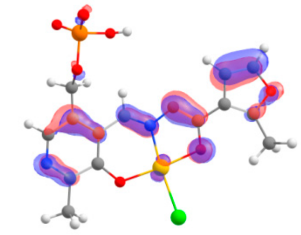	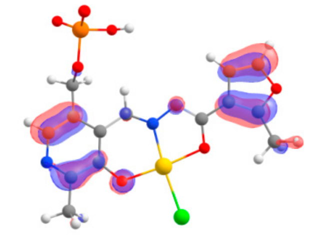	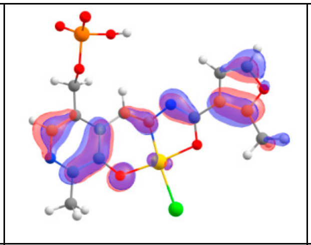	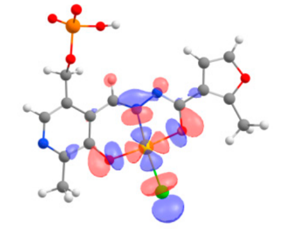	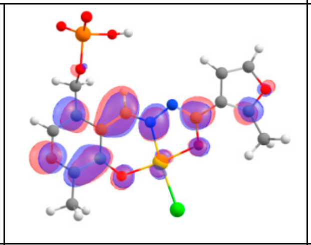
	HOMO-15(98)	HOMO-2(111)	HOMO-1(112)	HOMO(113)	LUMO(114)	LUMO+1(115)
AuClHL^0	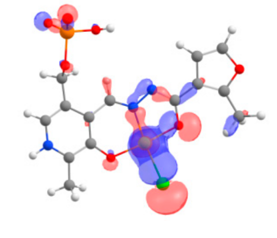	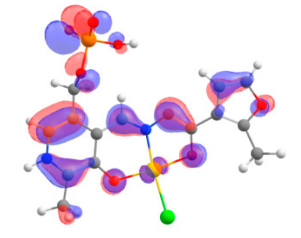	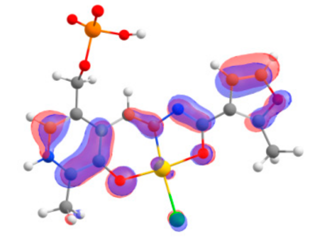	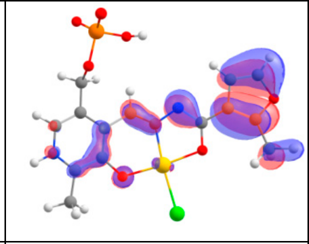	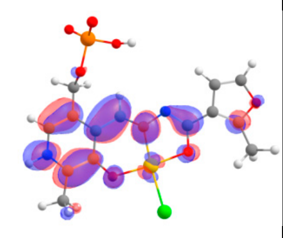	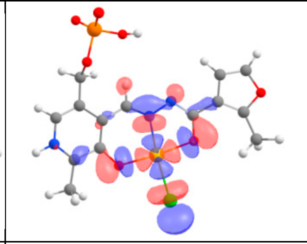
	HOMO-15(98)	HOMO-4(109)	HOMO-1(112)	HOMO(113)	LUMO(114)	LUMO+1(115)
AuClH$_2$L$^+$	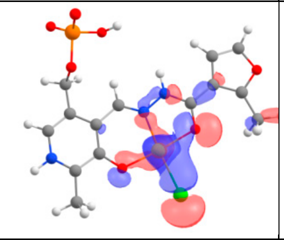	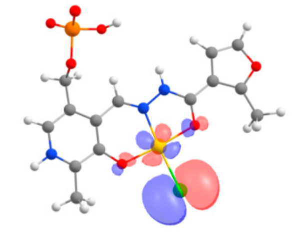	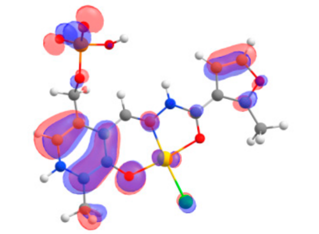	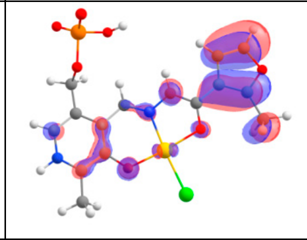	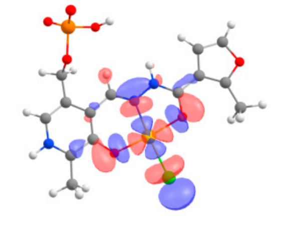	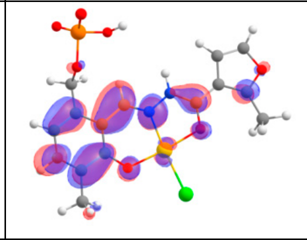
	HOMO-15(98)	HOMO-8(105)	HOMO-2(111)	HOMO(113)	LUMO(114)	LUMO+1(115)
**AuCl(PLP-F2H)**						
AuClL$^-$	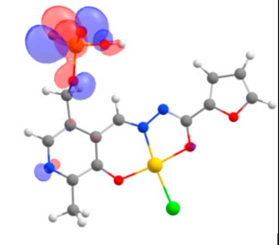	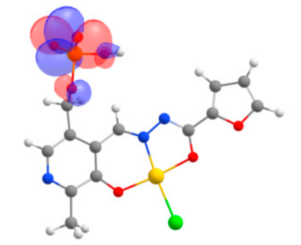	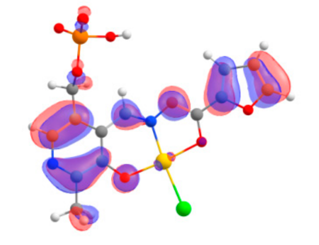	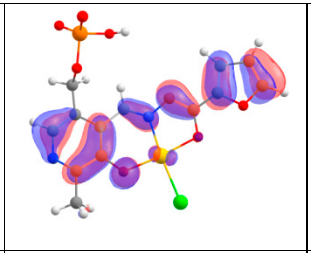	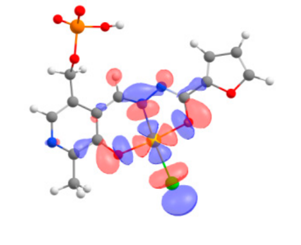	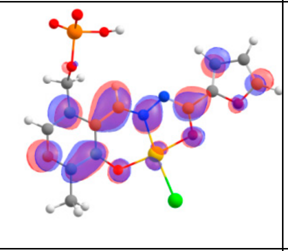
	HOMO-3(106)	HOMO-2(107)	HOMO-1(108)	HOMO(109)	LUMO(110)	LUMO+1(111)

$AuClHL^0$	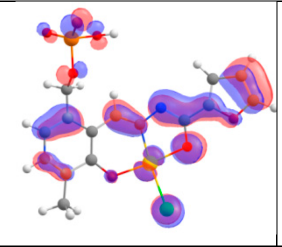	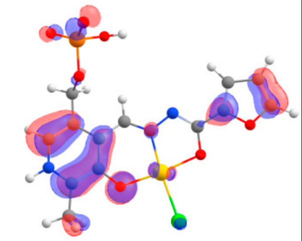	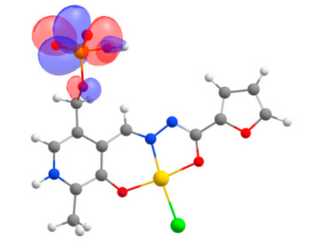	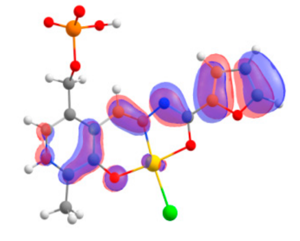	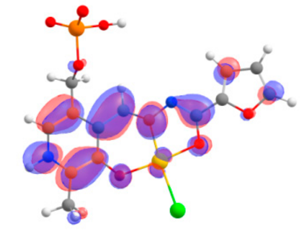	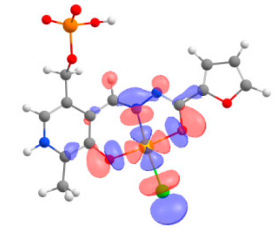
	HOMO-5(104)	HOMO-2(107)	HOMO-1(108)	HOMO(109)	LUMO(110)	LUMO+1(111)
$AuClH_2L^+$	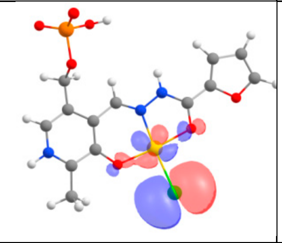	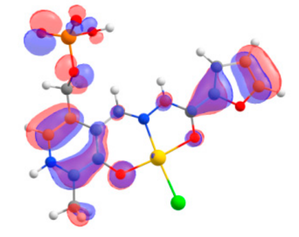	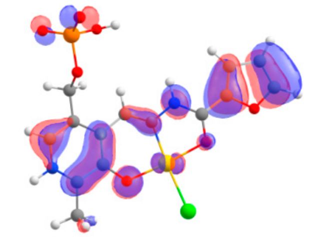	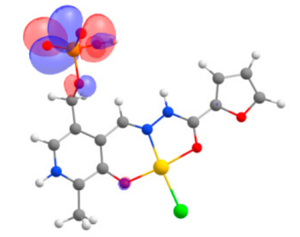	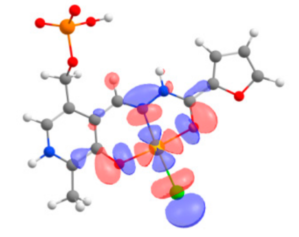	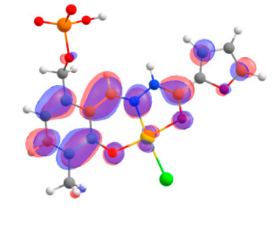
	HOMO-8(101)	HOMO-3(106)	HOMO-1(108)	HOMO(109)	LUMO(110)	LUMO+1(111)
**AuCl(PLP-T3H)**						
$AuClL^-$	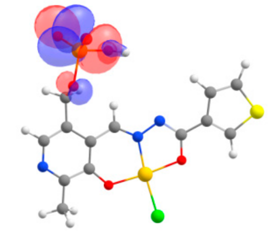	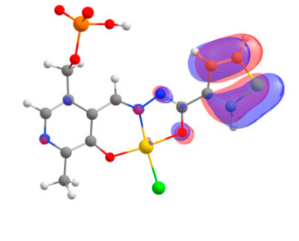	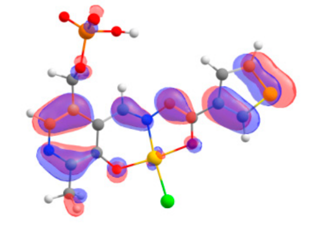	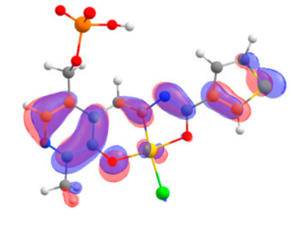	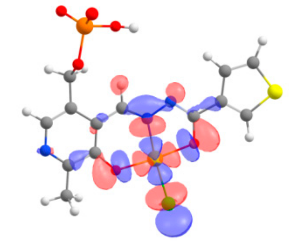	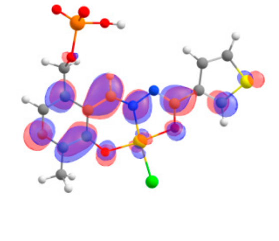
	HOMO-3(110)	HOMO-2(111)	HOMO-1(112)	HOMO(113)	LUMO(114)	LUMO+1(115)
$AuClHL^0$	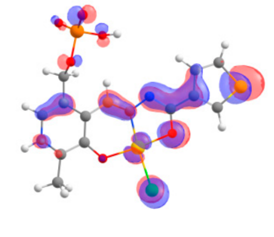	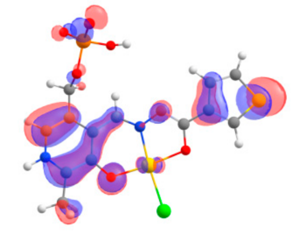	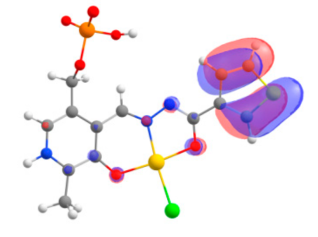	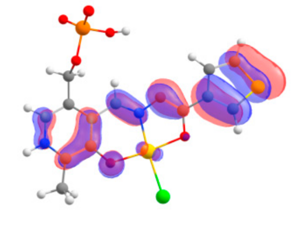	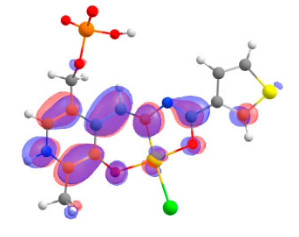	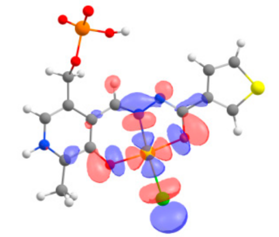
	HOMO-6(107)	HOMO-3(110)	HOMO-1(112)	HOMO(113)	LUMO(114)	LUMO+1(115)
$AuClH_2L^+$	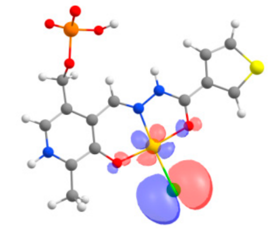	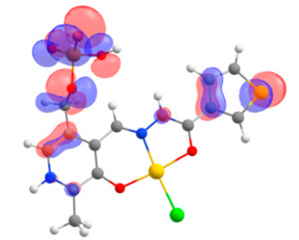	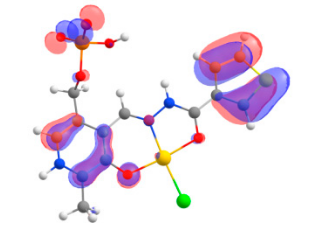	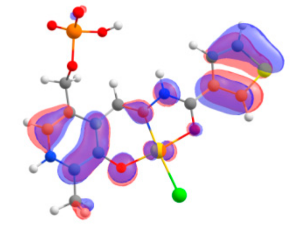	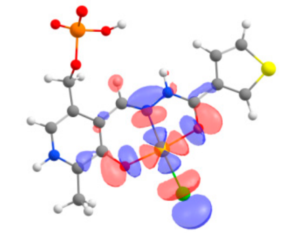	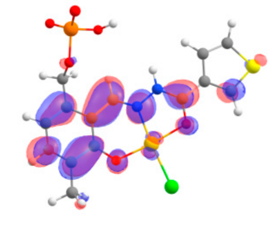

	HOMO-9(104)	HOMO-4(109)	HOMO-2(111)	HOMO-1(112)	LUMO(114)	LUMO+1(115)
**AuCl(PLP-T2H)**						
AuClL$^-$	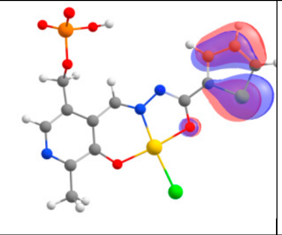	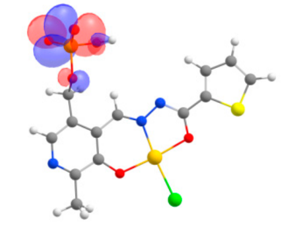	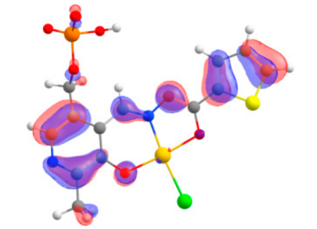	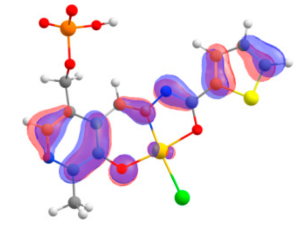	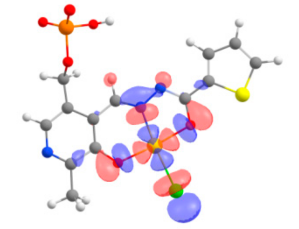	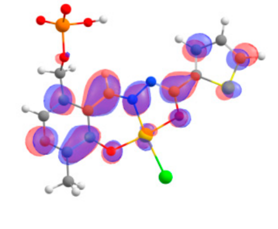
	HOMO-3(110)	HOMO-2(111)	HOMO-1(112)	HOMO(113)	LUMO(114)	LUMO+1(115)
AuCLHL^0	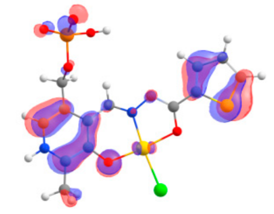	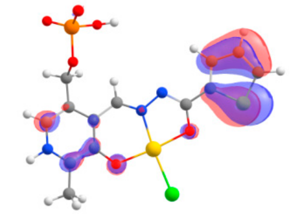	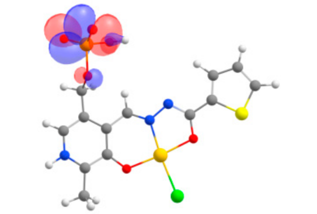	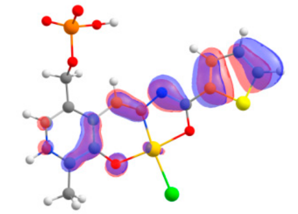	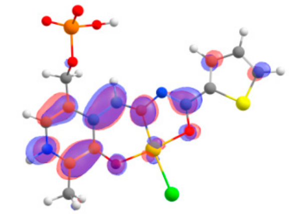	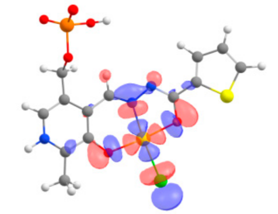
	HOMO-3(110)	HOMO-2(111)	HOMO-1(112)	HOMO(113)	LUMO(114)	LUMO+1(115)
AuClH$_2$L$^+$	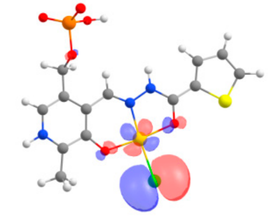	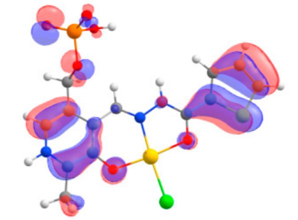	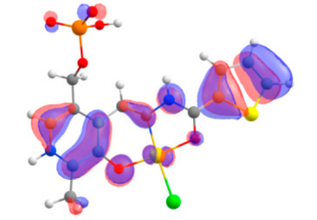	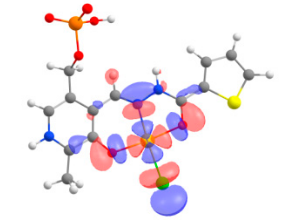	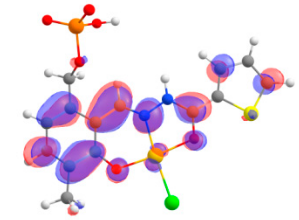	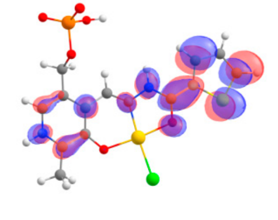
	HOMO-9(104)	HOMO-3(110)	HOMO-1(112)	LUMO(114)	LUMO+1(115)	LUMO+2(116)
**AuCl(PLP-INH)**						
AuClL$^-$	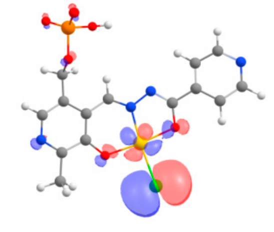	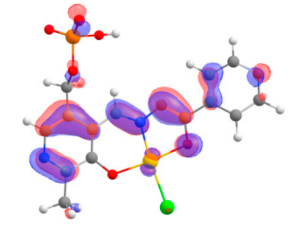	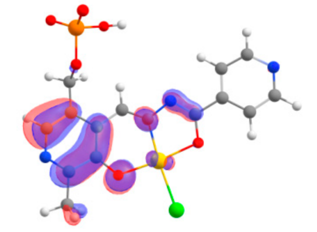	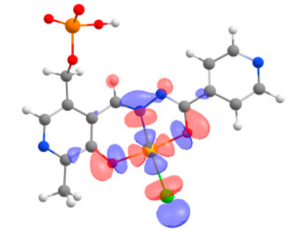	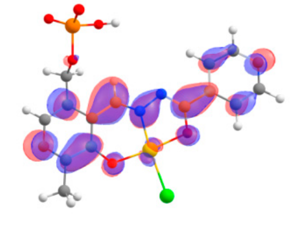	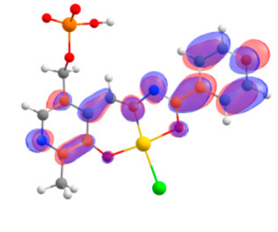
	HOMO-10(103)	HOMO-1(111)	HOMO(112)	LUMO(113)	LUMO+1(114)	LUMO+2(115)
AuClHL^0	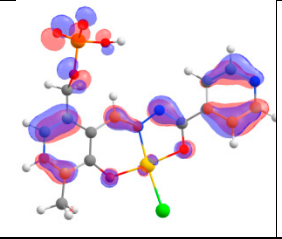	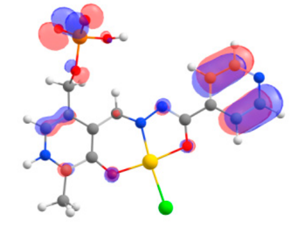	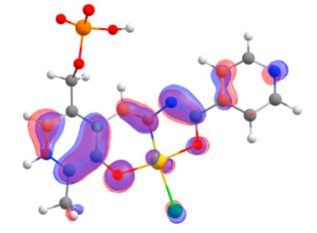	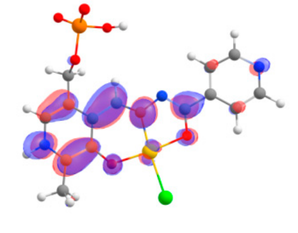	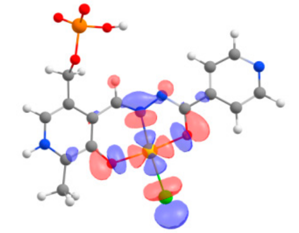	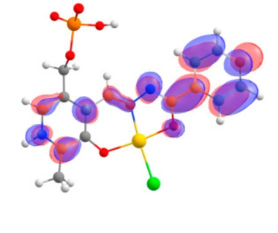

	HOMO-4(108)	HOMO-3(109)	HOMO(112)	LUMO(113)	LUMO+1(114)	LUMO+2(115)	
$AuClH_2L^+$	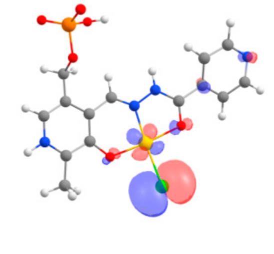	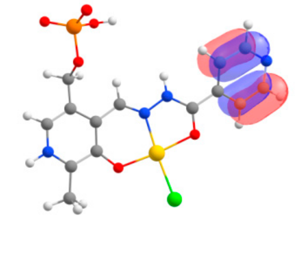	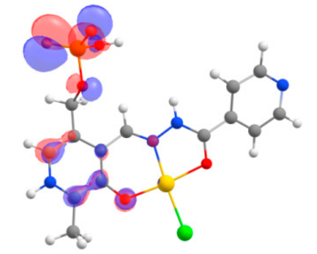	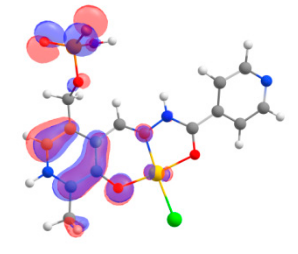	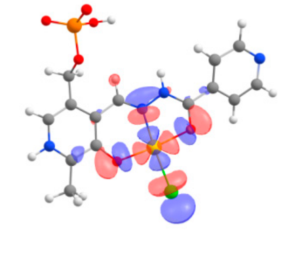	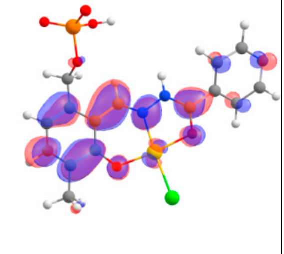	
	HOMO-9(103)	HOMO-4(108)	HOMO-2(110)	HOMO-1(111)	LUMO(113)	LUMO+1(114)	

Figure S2. Shape of selected frontier molecular orbitals. of gold(III) hydrazone complexes obtained by CAM-B3LYP calculations.

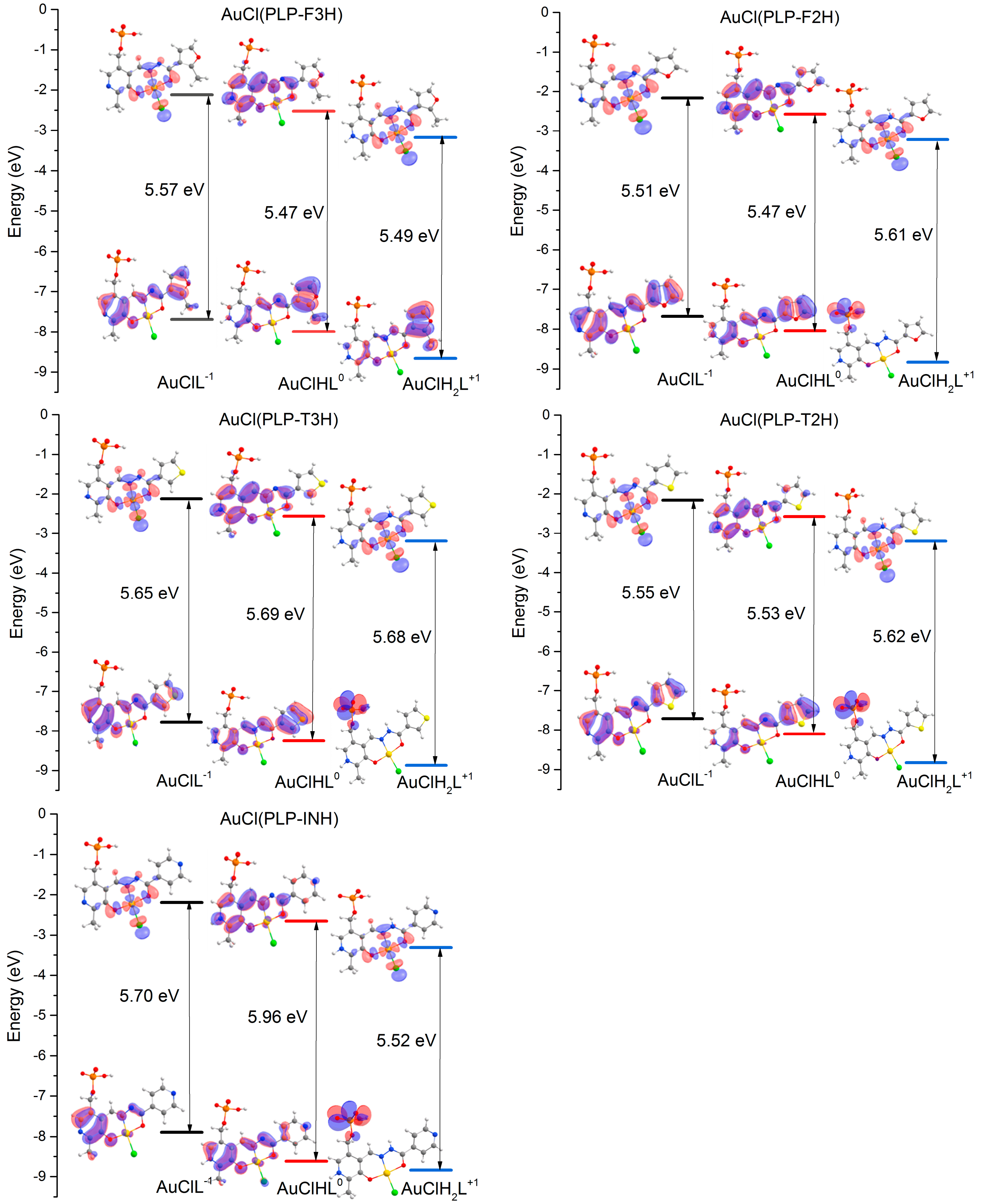
AuCl(PLP-F3H)
Energy (eV)
0
-1
-2
-3
-4
-5
-6
-7
-8
-9
5.57 eV
5.47 eV
5.49 eV
AuClL^{-1}
AuClHL^{0}
AuClH$_2$L^{+1}
AuCl(PLP-F2H)
5.51 eV
5.47 eV
5.61 eV
AuCl(PLP-T3H)
5.65 eV
5.69 eV
5.68 eV
AuCl(PLP-T2H)
5.55 eV
5.53 eV
5.62 eV
AuCl(PLP-INH)
5.70 eV
5.96 eV
5.52 eV

Figure S3. The HOMO/LUMO energy level diagram of gold(III) hydrazone complexes obtained by CAM-B3LYP calculations.

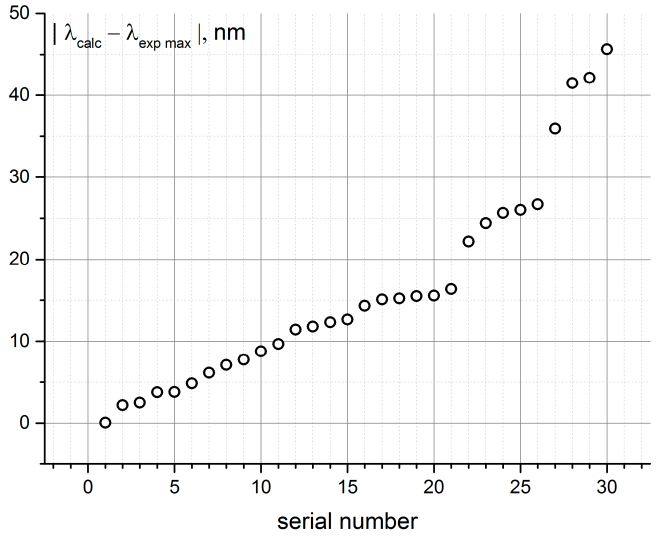

Figure S4. The ascending deviation $|\lambda_{cal} - \lambda_{exp\ max}|$ diagram for the considered excited states (see Table 4 from main text of the paper).
